# Ten years Diffusion Model for Conflict (DMC) tasks: Theoretical foundations, applications, practical recommendations, and open challenges

**DOI:** 10.3758/s13423-026-02878-8

**Published:** 2026-04-22

**Authors:** Markus Janczyk, Ian G. Mackenzie, Rolf Ulrich, Valentin Koob

**Affiliations:** 1 Psychological Research Methods and Cognitive Psychology, Department of Psychology, University of Bremen, Hochschulring 18, D-28359 Bremen, Germany; 2 Eberhard Karls University Tübingen, Tübingen, Germany

**Keywords:** DMC, Diffusion Model for Conflict tasks, Diffusion model, Conflict tasks, Simon, Flanker, Delta plots

## Abstract

A central question in cognitive psychology concerns how humans selectively attend to task-relevant information while ignoring task-irrelevant information. This question is frequently studied using conflict tasks, such as the Simon, Eriksen flanker, and Stroop tasks that require responses to relevant stimulus features while ignoring irrelevant and potentially conflicting features. All conflict tasks produce better performance in congruent trials where task-relevant features and irrelevant features match compared with when they mismatch in incongruent trials: the congruency effect. However, they differ markedly in the temporal dynamics of this congruency effect and accordingly in their RT distributions. The Diffusion Model for Conflict tasks (DMC) was introduced as a model to formally account for all distributional patterns in conflict tasks. It integrates dual-route theories of cognitive control into the framework of sequential sampling models by positing two superimposed, independent evidence accumulation processes: a linear one for controlled processing of the task-relevant information, and a pulse-like one for automatic processing of the task-irrelevant information, in which activation first increases until a maximum and then decreases again. This review summarizes DMC’s architecture, its core parameters, and its ability to account for various distributional patterns. We review and discuss applications of DMC across several psychological domains, and technical considerations such as parameter estimation and recovery. Limitations of the model are critically assessed, and fields of open research are outlined. Overall, DMC offers a general account of conflict processing. While a powerful tool for quantifying the dynamics of selective attention and cognitive control, there is still a limited standardization in its application, and more research is needed to extend DMC to other classes of conflict tasks.

**Supplementary Information:**

The online version contains supplementary material available at 10.3758/s13423-026-02878-8.

An important topic in cognitive psychology concerns whether and how humans can selectively attend to task-relevant information while ignoring task-irrelevant information in goal-directed behavior. Consider a driver intending to obey a red traffic light before proceeding straight through an intersection. A traffic light for right-turners may then switch to green. Although it is irrelevant to the driver’s intended action, this conflicting traffic light could automatically trigger a tendency to start driving straight. Such situations are frequently investigated in the laboratory with *conflict tasks*.

**Fig. 1:**
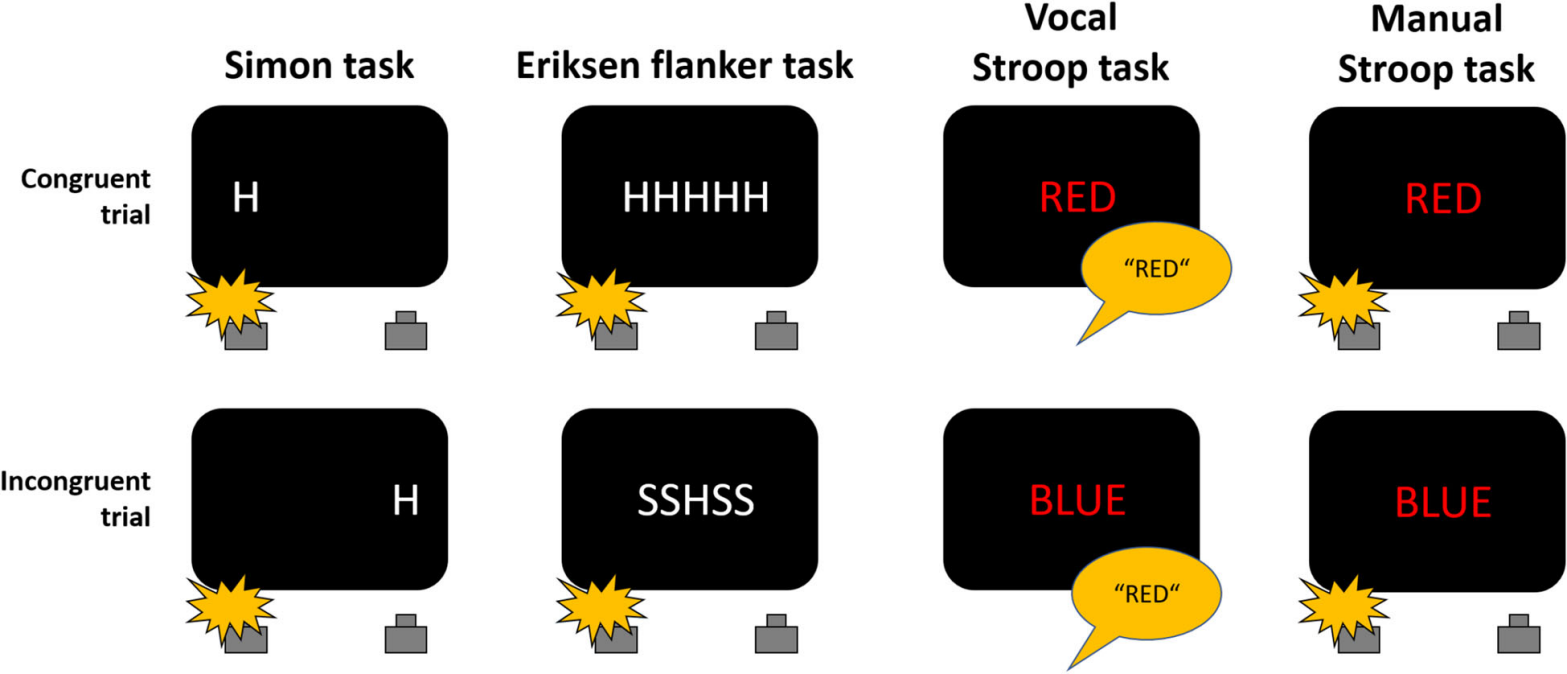
Illustration of the prototypical conflict tasks relevant to the present article. In the exemplary (visual) Simon and Eriksen flanker task, the letter H requires a left response while the letter S requires a right response. In the exemplary vocal Stroop task, participants name the font color, and in the exemplary manual Stroop task, the red font color requires a left response

The most-often used conflict tasks are illustrated in Fig. 1. They all exhibit a structural similarity: Participants are required to respond to task-relevant stimulus features and to ignore task-irrelevant ones. In the Simon task (Simon, 1969; see also Hommel, 2011; Leuthold, 2011, for reviews), a left/right key press is given to, for example, a letter’s identity, while its location is to be ignored. In the Eriksen flanker task (Eriksen & Eriksen, 1974), a left/right key press is given to the identity of a central target, while surrounding flankers are to be ignored. In the vocal Stroop task (MacLeod, 1991; Stroop, 1935), the font-color of a color word is to be uttered, while the color word itself is to be ignored, and in the manual Stroop task, a response to the font-color is given as a key press. While conflict tasks share this common structure, they differ in the specific type of conflict they are designed to elicit. In the Simon task, the conflict is between the task-irrelevant stimulus location and the spatially defined response. In the Eriksen flanker task, the conflict is between the central target and the surrounding flankers and, in addition, between the responses that are associated with each stimulus on the display. Finally, in the (vocal) Stroop task, the task-relevant font color and the response conflict with the task-irrelevant semantic meaning of the color word (see Hommel, 2011; Kornblum, Hasbroucq, & Osman, 1990; Kornblum & Lee, 1995, for more thorough discussions of conflict types).

Despite these differences, a *congruency effect* is typically observed: Response times (RTs) are shorter and fewer errors are made in *congruent trials*, where the task-relevant and the task-irrelevant features suggest the same response, compared with *incongruent trials*, where they suggest different responses. An explanation for this observation is that stimuli are processed along two different routes or channels. The *controlled route* processes the task-relevant stimulus feature in the instructed way, while the *automatic route* processes the task-irrelevant stimulus feature in an automatic, unconditional way.^1^ The resulting activations for one or the other response are merged and if, as in congruent trials, they indicate the same response, selection is faster compared with when they indicate different responses, as in incongruent trials (e.g., De Jong, Liang, & Lauber, 1994; Kornblum et al., 1990).

While the dual-route architecture has guided research on conflict tasks and congruency effects for decades, it does not specifically address the dynamics within and between the proposed routes. To address this, several formal models of conflict tasks have been developed over the past fifteen years (e.g., Heuer, Seegelke, & Wühr, 2023; Hübner, Steinhauser, & Lehle, 2010; Lee & Sewell, 2024; López & Pomi 2024; Miller & Schwarz, 2021; Ulrich, Schröter, Leuthold, & Birngruber, 2015; White, Ratcliff, & Starns, 2011). These models provide a deeper understanding of the precise dynamics underlying task-relevant and task-irrelevant processing, and they account not only for the congruency effect itself, but also for the full distribution of observed RTs. From this, for example, the temporal dynamics of the congruency effect can be analyzed, that is, whether a congruency effect becomes smaller or larger the longer the RTs are.

**Fig. 2:**
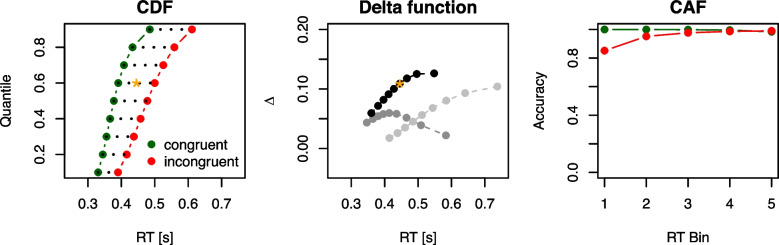
Illustration of an exemplary CDF, several delta functions, and a CAF. The *left panel* illustrates exemplary CDFs from a conflict task with quantile levels on the *y*-axis and the corresponding RT in seconds on the *x*-axis. The *dotted lines* highlight the differences between conditions for each quantile, that is, the values on the *y*-axis of a delta function. The 60% quantile level is marked with an *orange star*. The *middle panel* displays the delta function (*black dots*), derived from these CDFs. The *orange star* marks the point corresponding to the 60% quantile. Two additional (positively and negatively sloped) delta functions are visualized in *light gray* and *dark gray*, respectively (their corresponding CDFs are not shown). The *right panel* shows an example CAF with accuracy (*y*-axis) plotted as a function of RT bin (*x*-axis), separately for congruency conditions. To construct a CAF, all RTs of a congruency condition are binned into equally spaced bins and the accuracy per bin is then calculated. Note that we use seconds instead of milliseconds here, to be consistent with the time unit of DMC later in this review

## Purpose and outline of the present manuscript

This manuscript reviews the Diffusion Model for Conflict tasks (DMC; Ulrich et al., 2015), which has gained considerable popularity over the last ten years. One reason is that it is among the few models that generalize across different conflict tasks, offering a broad theoretical and modeling framework. The goal of this article is to provide a comprehensive review of DMC and its applications, accessible to readers both familiar and unfamiliar with the model. This, of course, comes with some deliberate choices about the scope of the article. First, it is about DMC. Other models, although certainly worthwhile and important, are only touched upon briefly. Exceptions are two models that have a direct relation to DMC (Lee & Sewell, 2024; López & Pomi, 2024). Second, it is not a tutorial on fitting DMC by providing annotated code examples. Code to fit DMC with the dRiftDM package in R is provided in Koob, Richter, Ulrich, and Janczyk (2025a).

We continue by outlining the key empirical effects that any general model of conflict tasks should be able to predict, followed by a description of DMC’s architecture and parameters. Readers familiar with DMC may want to skip this section, although we touch upon some details (e.g., the effects of parameter variations) that may come with new insights. The second section then considers two models for conflict tasks that bear a conceptual relation to DMC, which is particularly relevant for those who are interested in critiques of DMC. The third section reviews applications of DMC across various fields of psychology, and the fourth section turns to more technical issues, including parameter estimation, recovery, and mathematical peculiarities of certain parameters. This section is most interesting for researchers already using DMC who want to learn more about potential issues and recent developments in practical work with DMC. Finally, the fifth section focuses on (potential) limitations of DMC and on future research needed to validate and expand DMC, and is followed by a brief conclusion with some general remarks.

### Critical effects to be accounted for

Despite its importance and prevalence in the empirical literature, the standard congruency effect is only a rough description of performance across the different conflict tasks. More nuanced analyses consider the entire RT distributions of correct (and incorrect) responses and analyze the congruency effect as a function of RT level with so-called *delta functions* (De Jong et al., 1994). To construct a delta function, the cumulative distribution functions (CDFs) of correct RTs are computed separately for congruent and incongruent trials. The RT difference between these two CDFs at a given quantile level reflects the size of the congruency effect at a specific RT level. For example, the difference at the 20% quantile level represents a difference between relatively fast congruent and incongruent responses, while the difference at the 80% quantile level represents the congruency effect at rather slow responses. The actual delta function plots these RT differences at each quantile level against their mean. In other words, the delta function is the congruency effect as a function of RT. The left and middle panel of Fig. 2 visualize exemplary CDFs and the corresponding delta function.

Roughly, two different patterns can occur (see Fig. 2, middle panel): The congruency effect can either increase or decrease with longer RTs, and we refer to them as *positively* and *negatively sloped* delta functions. The former is the standard pattern predicted by many RT models (see Wagenmakers & Brown, 2007). From a mathematical perspective, they result when the variance of an RT distribution increases with longer mean RTs. The Eriksen flanker task and the Stroop task exhibit this pattern (e.g., Hübner & Töbel, 2019; Mittelstädt, Mackenzie, Koob, & Janczyk, 2023; Pratte, Rouder, Morey, & Feng, 2010; Ulrich et al., 2015), although the precise shape of their delta functions can vary with different stimuli and the interval between the onset of task-relevant and task-irrelevant stimuli (Hübner & Töbel, 2019; Mackenzie, Mittelstädt, Ulrich, & Leuthold, 2022; Pratte, 2021). *Negatively sloped* delta functions, where the congruency effect diminishes with longer RTs, result when the variance of an RT distribution decreases with longer (mean) RTs. This pattern occurs in the visual, horizontal Simon task (Hübner & Töbel, 2019; Koob, Mackenzie, Ulrich, Leuthold, & Janczyk, 2023b; Mittelstädt et al., 2023; Pratte et al., 2010; Servant, Montagnini, & Burle, 2014; Ulrich et al., 2015; see also Proctor, Miles, & Baroni, 2011), and the congruency effect may even reverse at the longest RTs. Any general mathematical model of conflict tasks should be able to predict both positively and negatively sloped delta functions in addition to the simple congruency effect.

In addition, there are two further observations that a model of conflict tasks should account for. The first result concerns accuracy (or error rate). When accuracy is calculated separately for equally sized RT bins, results usually differ between congruent and incongruent conditions. While accuracy is high and relatively constant across RT bins for the congruent condition, accuracy increases with RT bin for the incongruent condition. For the Eriksen flanker task, this effect has also been highlighted by White et al. (2011, p. 212), who state that “error RTs were faster than correct RTs for incongruent trials, suggesting that flanker interference is strongest early in the trial”. This differential effect is often captured and visualized with *conditional accuracy functions* (CAFs; Stins, Polderman, Boomsma, & de Geus, 2008; see the right panel of Fig. 2 for a visualization). The second result is that the congruency effect’ size sometimes remains fairly stable when a task-relevant stimulus feature is manipulated. This was demonstrated by Servant et al. (2014) for the Eriksen flanker and the Simon task by varying the color saturation of the targets (see also Stafford, Ingram, & Gurney, 2011, for a similar finding with the Stroop task). While this manipulation influenced the overall RT level by approximately 50 ms, the congruency effect itself remained fairly unchanged.

**Fig. 3:**
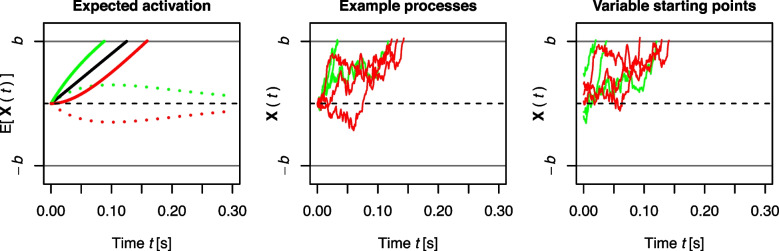
Illustration of automatic and controlled processing in DMC. The *left panel* illustrates the expected activations as a function of time *t*. The *black solid line* represents controlled processing. The *dotted green* and *red lines* represent automatic processing in congruent and incongruent trials, respectively. In congruent trials, automatic processing provides activation toward the upper boundary *b* (a correct response), and in incongruent trials it provides activation toward the lower boundary $-b$ (an incorrect response). The *solid green* and *red lines* are the sum of both activations for congruent and incongruent trials. The *middle panel* shows examples for noisy evidence accumulation trajectories when Brownian motion is added. The *right panel* visualizes examples when variability in the starting point of the evidence accumulation processes is incorporated

### The standard diffusion model and the architecture of DMC

DMC is a version of the diffusion model (DM) that was popularized in psychological research by Ratcliff (1978; see also Ratcliff, Smith, Brown, & McKoon, 2016; Voss, Nagler, & Lerche, 2013, Voss, Voss, & Lerche, 2015, for reviews) as a process model for two-choice RT tasks.

#### The standard diffusion model

Its core idea is that responding to a stimulus involves two components: a decision process, reflecting response selection, and a residual process, reflecting perceptual and motor time-requirements. In the standard DM, the decision component is a noisy evidence accumulation process over time. The systematic component of the evidence accumulation is a straight line, representing linearly increasing activation with a slope termed the drift rate $\mu$. The noise is incorporated by adding a Brownian motion that leads to random fluctuations in evidence. This noise is typically multiplied by the diffusion coefficient $\sigma$, which mainly serves to scale the entire accumulation process and is almost always a fixed (scaling) constant. Formally, we can write the current evidence or activation state of the decision process at time *t* as

1 $$\begin{aligned} \textbf{X}(t) = \mu t + \sigma \textbf{B}(t) \, . \end{aligned}$$

Here, $\textbf{X}(t)$ represents the accumulated evidence at time *t*. The term $\mu t$ reflects the systematic component of the decision process, and the longer the decision process runs, the more evidence is accumulated. The Brownian motion, $\textbf{B}(t)$, is the random component that adds noise to evidence accumulation (see the middle panel of Fig. 3 for a visualization of this in the context of DMC).^2^ The evidence accumulation continues until one of two (symmetric) boundaries *b* or $-b$ is exceeded. This indicates that sufficient evidence has been accumulated, and a response is considered selected. The time *t* needed to reach a boundary is then the decision time.

Basically, the upper and lower boundaries *b* and $-b$ code the two possible responses, highlighting that such DMs mainly apply to tasks with two responses. However, often boundaries are *accuracy-coded* in practice. Assuming that the drift rate drives the process toward the correct response, the respective response is coded via the upper boundary, while the lower boundary codes other, incorrect responses. This coding thus partly allows handling tasks with more than two stimuli and responses, often assuming that the drift rate is equal for each stimulus/response. However, with accuracy-coding, it cannot be distinguished which particular response was chosen as the “wrong” response. Thus, the extension of DMs to tasks with multiple responses is limited to some degree.

The duration of perceptual and motor processes is summarized in the non-decision time, which is added to the decision time to yield RTs proper. In essence, then, the DM provides a process model that decomposes RTs into a set of parameters for the decision and non-decision components of simple tasks (for more in-depth information on the interpretation and validation of the standard DM, see Voss, Rothermund, & Voss, 2004).

#### The architecture of DMC

DMC translates dual-route models into the DM framework. It starts with assumptions regarding the time course of expected activation within the controlled and automatic route in response to the task-relevant and task-irrelevant stimulus features, respectively (e.g., the central target and the flankers in the Eriksen flanker task). We denote the current activation/evidence for the controlled and automatic route at time *t* as $\mathbf {X_c}(t)$ and $\mathbf {X_a}(t)$, respectively. Within the *controlled route*, activation is assumed to increase linearly, as in the standard DM (see Eq. 1). Denoting the slope of this increase as $\mu _c$, the expected activation is then

2 $$\begin{aligned} \mathbb {E}[\mathbf {X_c}(t)]=\mu _c t\,. \end{aligned}$$

This is represented by the straight black line in the left panel of Fig. 3. Given that, in technical terms, drift rates reflect the momentary rate of change (Cox & Miller, 1965; Schwarz, 2022), the controlled drift rate $\mu _c$ can be seen as the first derivative with respect to time *t*, that is, the slope of $\mathbb {E}[\mathbf {X_c}(t)]$ at each time point *t*:

3 $$\begin{aligned} \mu _c(t) = \frac{d\mathbb {E}[\mathbf {X_c}(t)]}{dt}=\mu _c\,. \end{aligned}$$

The expected activation within the *automatic route*, $\mathbb {E}[\mathbf {X_a}(t)]$, is assumed to follow a “pulse-like” form: It first increases to a maximum and then gradually fades to zero as time *t* increases. This pulse-like time course of activation is conceptually and empirically consistent with two views (for more details and references, please see Ulrich et al., 2015, p. 153): First, activation in the automatic route decays passively (Hommel, 1994) after some time, as it is (mainly) the result of task-irrelevant stimulation. Second, activation in the automatic channel is actively inhibited and suppressed, maybe because it is potentially harmful to the decision (Ridderinkhof, 2002a). Several potential candidate functions match this general form, and DMC uses a (rescaled) Gamma distribution function to describe the expected automatic activation:

4 $$\begin{aligned} \mathbb {E}[\mathbf {X_a}(t)]=A e^{-\frac{t}{\tau }}\cdot \left[ \frac{t e}{(a-1)\tau }\right] ^{a-1}\,. \end{aligned}$$

The drift rate of the automatic process, reflecting its momentary rate of change, is the first derivative of $\mathbb {E}[\mathbf {X_a}(t)]$ with respect to time. Note that the resulting equation now depends on *t* and hence the drift rate $\mu _a(t)$ is said *time-dependent*:

5 $$\begin{aligned} \begin{aligned} \mu _a(t)&=\! \frac{d\mathbb {E}[\mathbf {X_a}(t)]}{dt} =\! A e^{-\frac{t}{\tau }} \cdot \left[ \frac{t e}{(a-1) \tau } \right] ^{a-1} \cdot \left[ \frac{a-1}{t} - \frac{1}{\tau } \right] \,. \end{aligned} \end{aligned}$$

As will be discussed in more detail below, *A* determines the maximum amplitude of this function while the shape and scale parameters, *a* and $\tau$, jointly determine the time point of this maximum, which is achieved at $t_{\text {max}}=(a-1)\tau$. Automatic activation aligns with the controlled activation in congruent trials, but opposes it in incongruent trials. With $\mu _c>0$, *A* then has a positive sign in congruent and a negative sign in incongruent trials. The left panel of Fig. 3 visualizes examples of automatic activation as dotted green and red lines.

Finally, the overall decision process is the sum of the activations within the controlled and the automatic route, and the expected time course is shown as the solid green and red lines in the left panel of Fig. 3. When adding the Brownian motion as noise as well, the activation of the overall decision process in DMC can be written as follows (which represents an extension of Eq. 1 of the standard DM):

6 $$\begin{aligned} \begin{aligned} \textbf{X}(t)&=\! \mathbf {X_c}(t) + \mathbf {X_a}(t) =\! \mu _c t + A e^{-\frac{t}{\tau }}\left[ \frac{t e}{(a-1)\tau }\right] ^{a-1} + \sigma \textbf{B}(t) \,. \end{aligned} \end{aligned}$$

As in the standard DM, a response is selected once the overall decision process $\textbf{X}(t)$ reaches one of the decision boundaries *b* or $-b$. In DMC, boundaries are typically accuracy-coded, that is, the upper and lower boundaries code correct and incorrect responses, respectively. This partly allows for handling tasks with more than two stimuli and responses. In general, the architecture of DMC’s decision process gained support from a study considering neuronal measures and DMC simultaneously (Servant, White, Montagnini, & Burle, 2016).

While Fig. 3 summarizes the core assumptions of DMC, further model parameters help to make it adequately predict RTs in conflict tasks. First, time-requirements outside the decision process are captured by a non-decision time that is added to the decision time. Originally, this non-decision time within DMC is drawn from a normal distribution with expected mean *t*0 and a standard deviation $S_{t0}$, but other distributions for the non-decision time are possible, of course.

So far, we assumed that evidence accumulation starts at a starting point of zero, that is, $\textbf{X}(0)=0$. This is, however, not necessary (see the right panel of Fig. 3). Specifically, Ulrich et al. (2015) introduced DMC with a random variability of the starting point, which is drawn from a symmetric Beta distribution with a parameter $\alpha$ and centered in between the boundaries. Accordingly, evidence accumulation sometimes starts closer to the upper boundary, and sometimes closer to the lower boundary, but on average at zero without a systematic bias. Such additional sources of variability are common for DMs in general (e.g., Ratcliff, 2013; Ratcliff & Rouder, 1998) and they help account for specific data patterns that are not well captured otherwise (e.g., fast errors in incongruent trials; see also the later Section “Fast errors and variability of the starting point’’).

### DMC’s parameters and their effects on model predictions

Having introduced the basic architecture and core assumptions of DMC, we now turn to its parameters in more detail. To this end, we will cover the interpretation of DMC’s parameters and their influence on RT levels, accuracy, and delta functions.

#### Interpretation of DMC’s parameters

All (eight) parameters of DMC are summarized in Table 1. We cover the interpretation of standard DM parameters only briefly, as they are covered elsewhere (e.g., Ratcliff et al., 2016; Ratcliff & Tuerlinckx, 2002; Voss et al., 2013). Readers new to the field of DMs find a longer elaboration in the Electronic Supplement A.

**Table 1:** Summary of DMC’s eight parameters

Parameter	Description
*b*	Decision boundary
$\mu _c$	Drift rate of controlled processing
*t*0	Mean of the normally distributed non-decision time
$S_{t0}$	Standard deviation of the normally distributed non-decision time
$\alpha$	Shape parameter for the starting point (Beta distribution)
*A*	Amplitude of task-irrelevant processing
$\tau$	Scale parameter of task-irrelevant processing (Gamma distribution)
*a*	Shape parameter of task-irrelevant processing (Gamma distribution)

**Fig. 4:**
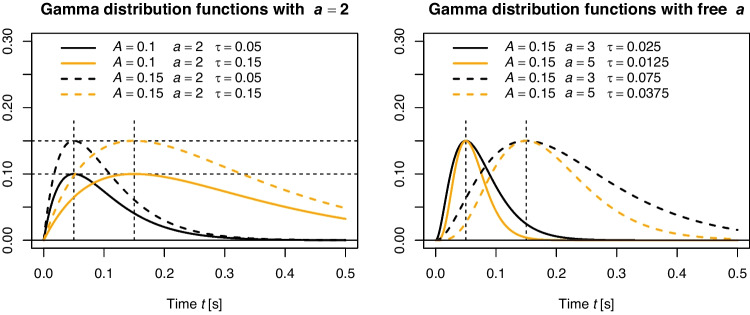
Illustration of the rescaled Gamma distribution function. The *left panel* visualizes examples for two levels of *A* and $\tau$, when fixing the shape parameter to $a=2$. In this case, the scale parameter $\tau$ directly determines the time point of the maximum. In the *right panel*, Gamma distribution functions with two different peaks are illustrated and one can see that *a* and $\tau$ jointly determine the peak with $t_\text {max}=(a-1)\tau$

#### Parameters shared with the standard DM

We begin with the parameters that DMC shares with the standard DM. The *boundary* *b* reflects how much evidence is needed until a response is made and it has opposite effects on RTs and error rate: Lower values yield shorter mean RTs and more errors. Hence, *b* captures differences in the speed-accuracy settings (Lerche & Voss, 2018; Liesefeld & Janczyk, 2019, 2023) and it is often taken to reflect an individual’s response style (i.e., more conservative vs. daring; Voss et al., 2004; see also Mittelstädt, Miller, Leuthold, Mackenzie, & Ulrich, 2022, for a study with DMC).

The (time-independent) *drift rate* $\mu _c$ is “the mean amount of information accumulated per unit of time” (Ratcliff & Rouder, 1998, p. 348). A larger drift rate yields shorter RTs and fewer errors, and it has been linked with task difficulty (Voss et al., 2004), and intelligence (Lerche et al. 2020; Schmiedek, Oberauer, Wilhelm, Süß, & Wittmann, 2007; Schubert, Frischkorn, Hagemann, & Voss, 2016). Within DMC, the drift rate $\mu _c$ reflects the speed of controlled processing of the task-relevant stimulus feature.

The *non-decision time* *t*0 and its standard deviation $S_{t0}$ represent the duration (and variability) of the non-decision processes, such as perception and motor execution. Increasing the non-decision time and its variability only increases the mean and the variance of RTs, but does not affect error rates, as these parameters do not interact with the central decision process. Often, the variability parameter $S_{t0}$ is less important than *t*0, and its primary purpose is to improve the model fit (Boehm et al., 2018; Lerche & Voss, 2016).

When *starting point variability* is included in DMC, starting points are drawn from a Beta distribution with shape parameter $\alpha$ (see Electronic Supplement B for more details). Starting point variability basically reflects random biases that are present even before the actual decision process begins. Usually, this parameter is also not of central theoretical interest, but is primarily used to predict fast errors and thereby improve model fit. Indeed, removing starting point variability typically impairs DMC’s ability to fit data of conflict tasks (see, e.g., Janczyk, Mackenzie, & Koob, 2025).

#### Parameters unique to DMC

We now turn to the DMC-specific parameters, which concern the automatic route’s activation. The *amplitude* *A* represents the peak level of automatic activation and can roughly be interpreted as the strength of this activation. However, its meaning slightly varies based on one’s theoretical perspective on *why* activation declines after reaching its maximum. If passive decay (Hommel, 1994) is considered the sole cause, then the interpretation of the strength of activation remains unchanged. However, suppose one posits that inhibition plays a role (Ridderinkhof, 2002a). In that case, *A* reflects a combination of the task-irrelevant stimulus feature’s potential to induce activation and the ongoing inhibition that counteracts this buildup. Thus, *A* indicates the maximum activation within the automatic channel, which may be lower than the theoretical activation level induced by the stimulus in the absence of inhibition. DMC itself is silent about this, but we will come back to this further below when discussing the model introduced by López and Pomi (2024).

The *shape parameter* *a* and the *scale parameter* $\tau$ of the Gamma distribution function jointly determine the time of maximum automatic activation, $t_{\text {max}}=(a-1) \tau$, and the exact shape of the function. Mathematically, *a* is any real positive number. Still, it is essential that $a > 1$ for DMC, because otherwise, the function takes on a shape that deviates from the desired pulse-like form. Often, even the constraint $a=2$ is used to reduce model complexity, and in this case, the time point of maximum activation is $t_{\text {max}}=\tau$. This is the original way DMC was parameterized (Ulrich et al., 2015). The left panel of Fig. 4 illustrates several examples for this situation with two values of $\tau$ and *A*. If both *a* and $\tau$ vary, the shape of the Gamma distribution function varies slightly with *a* for a fixed peak time and becomes narrower with increasing values of *a*. This is illustrated for two peaks and examples for the resulting values of *a* and $\tau$ in the right panel of Fig. 4. In the literature, there is some heterogeneity about whether *a* is fixed at 2 or not (e.g., Evans & Servant, 2022; Mittelstädt et al., 2023; Servant et al., 2016; Ulrich et al., 2015), and the question about whether one should fix *a* or not will be addressed in the later section “Technical aspects and recommendations”.

If taking a suppression stance, several measures might be derived from the Gamma distribution function to index the strength of inhibition that acts on the activation. For example, Servant, van Wouwe, Wylie, and Logan (2018) derived what they called *strength of inhibition* by calculating the time difference between $t_{\text {max}}$ and the “latency at which 90% of the automatic activation has been emitted” (p. 28). Such measures may offer a helpful interpretation. However, they require both *a* and $\tau$ to be estimated freely from empirical data; otherwise, with *a* fixed, they essentially carry the same information as $\tau$.

#### Effects on model predictions

**Fig. 5:**
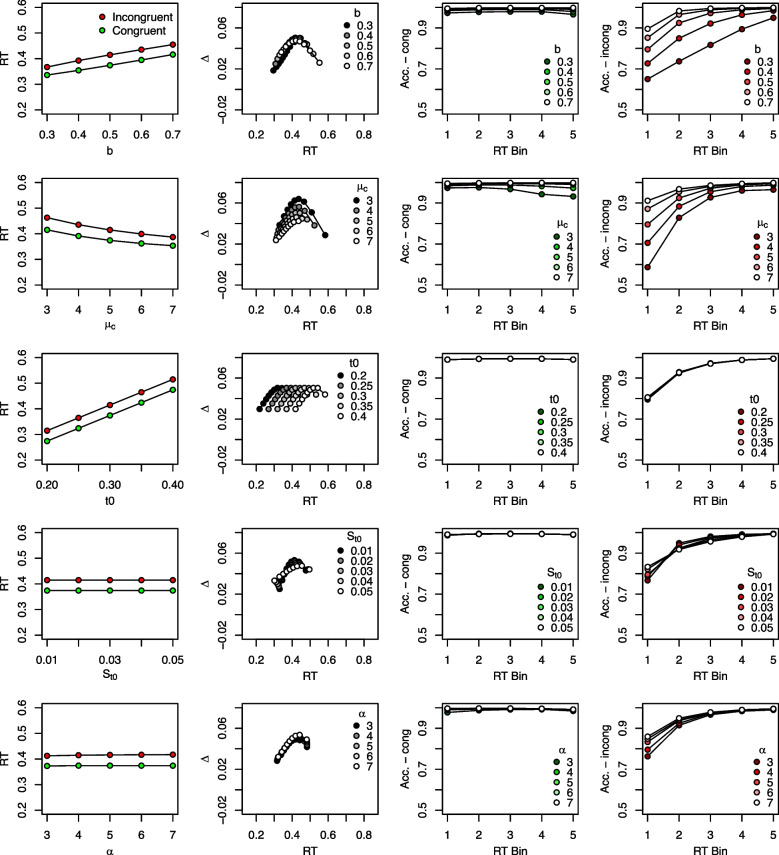

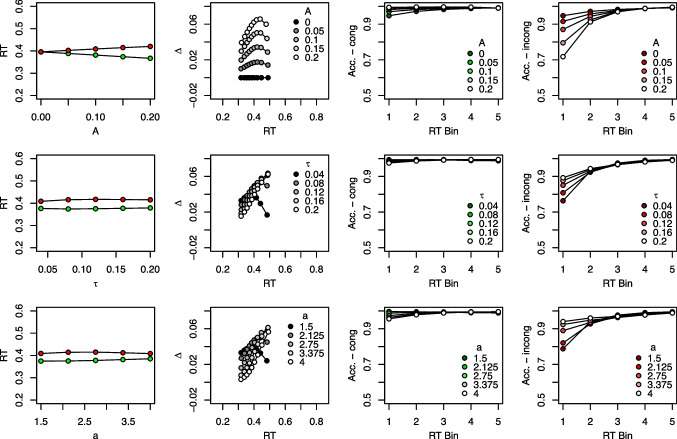
Illustration of DMC’s predictions as a function of each parameter. Each panel shows how DMC’s predictions change when one parameter is varied. Each parameter is represented in a separate row. The first column displays mean correct RTs, with the *x*-axis coding the respective parameter value. The second column displays delta functions and the parameter values are coded in *shades of gray*. The third and fourth column displays CAFs, separately for congruent and incongruent conditions, respectively (see also the *y*-axis label). This separation is not typical (see Fig. 2), but allows a better presentation in the present case. In the respective panels, the parameter values are coded in shades of *green* and *red*

In the previous section, we already touched upon the effects of parameters on RTs and error rates that are not unique to DMC. We now examine the influence of all DMC parameters in more detail, with a particular focus on the predicted congruency effects, delta functions, and CAFs. Figure 5 provides a summary of the model predictions when varying each parameter systematically. The first column of this figure presents mean correct RTs for both congruent and incongruent trials. The second column displays delta functions, and the third and fourth columns present CAFs, separately for congruent and incongruent trials, respectively. The values taken for the non-varied parameters were $b = 0.5$, $\mu _c = 5$, $t0=0.3$, $S_{t0}=0.03$, $\alpha = 4$, $A=0.15$, $\tau = 0.07$, and $a=2$, and were inspired by the parameter estimates of Ulrich et al. (2015).^3^ Please note also that the following effects of each parameter hold, of course, only for the particular values of the other parameters. To interactively explore the model predictions more flexibly, readers can use one of two ShinyApps: One is hosted online at https://bit.ly/dmc_shinyapp, and uses the |dRiftDM| R package (Koob et al., 2025a). Another is provided by the |DMCfun| R package (Mackenzie & Dudschig, 2021). After loading the package, run |dmcSimApp()|. Note, however, that the underlying packages differ in their scaling.^4^

We will discuss parameters as they are listed in Table 1. In general, Fig. 5 shows that DMC can predict data patterns typical for conflict tasks: It can predict an overall congruency effect (see the first column), positively and negatively sloped delta functions (second column), high and rather constant accuracy in congruent trials (third column), and lower accuracy for the first few RT bins in incongruent trials (fourth column).

#### Parameters shared with the standard DM

We begin with the parameters common to other DMs as well. Increasing the *boundary* *b* lengthens RTs, but does not much affect the overall congruency effect. It also yields more negatively sloped delta functions, which remain similar in their vertical level. In addition, accuracy is increased, in particular in the incongruent condition. Increasing the *controlled drift rate* $\mu _c$ decreases mean RTs while keeping the overall congruency effect largely unchanged. However, there is a slight tendency for smaller congruency effects with higher values of $\mu _c$. As mentioned above, fairly stable congruency effects when the task-relevant stimulus feature was manipulated have been observed (Servant et al., 2014). We will return to this toward the end of this section again. Increasing $\mu _c$ also lowers the vertical level of the delta function and causes it to become more positively sloped.

Higher values of $\mu _c$ increase accuracy, in particular in the incongruent condition. DMC also predicts lower accuracy for higher RT bins in congruent trials when $\mu _c$ is small (see also the predictions for small *b* values for a similar tendency), but this is not a typical empirical observation, and usually accuracy remains at a high level for the congruent condition. The reason for this prediction is that the impact of the beneficial automatic process diminishes over time in congruent trials.

Increasing the *non-decision time* *t*0 lengthens mean RTs, but does not affect the mean congruency effect or the shapes of the delta function and the CAFs. Increasing the *standard deviation of the non-decision time*, $S_{t0}$, has only marginal effects: mean RTs and the congruency effect remain unchanged, while the delta function and the CAF for incongruent trials become slightly less pronounced. Finally, variations in $\alpha$, that is, in the variance of the *starting point distribution*, have little effect on the mean congruency effect, the delta function, or the CAF in congruent trials. Its primary influence is on the CAFs in incongruent trials, which become more pronounced as $\alpha$ increases. This is why including $\alpha$ can improve model fit: It allows for fine-tuning of predicted accuracy in the fast RT bins, particularly for incongruent trials. We will revisit this point later when discussing modeling recommendations.

In essence, the parameters that are closely related to those in the standard DM behave similarly in DMC with respect to mean RTs and overall accuracy. However, it is noteworthy that especially *b* and $\mu _c$ also have a substantial impact on delta functions and the CAFs. This is because both parameters influence the overall duration of the decision process: higher values of *b* and lower values of $\mu _c$ lead to longer evidence accumulation. As a result, they affect how strongly the automatic process can exert its influence and, ultimately, how conflict unfolds throughout a trial.

#### Parameters unique to DMC

We now turn toward the DMC-specific parameters, namely those pertaining to the automatic process. The mean congruency effects become clearly larger with increasing values of *A*. Such larger amplitudes of the automatic process may, for example, result when the number of flankers presented at each side of the target increases. As the congruency conditions differ only in the sign of *A*, it is clear that no congruency effect at all occurs with $A=0$. The strong influence of *A* on the congruency effect is also reflected in the delta functions and the CAFs. With an increasing *A*, the steepness of the delta function and its overall vertical level become more prominent. The general shape, however, remains unaffected. With respect to the CAFs, slightly higher accuracy occurs in the first few RT bins with higher values of *A* in congruent trials. The opposite is true for incongruent trials, where, accuracy drops drastically for the first few RT bins with higher values of *A*.

The mean congruency effect in RTs remains fairly stable when varying $\tau$, although it tends to decrease with small values for $\tau$. Importantly, $\tau$ has a dramatic effect on the delta function: While the slope is negative with small values, it quickly becomes more positive as $\tau$ increases. This is indeed a main feature of DMC, which selectively changes the slope of a delta function by varying only one parameter’s value. Remember that, with $a=2$, $\tau$ directly indicates the time of maximum automatic activation, $t_\text {max} = (2-1) \tau = \tau$. Thus, an early peak of the automatic activation yields a negatively sloped delta function, while a late peak yields a positively sloped delta function. Indeed, automatic activation due to the irrelevant feature conceivably happens earlier in the Simon task than, for instance, in the Eriksen flanker task. In the former, the location of the stimulus directly activates the spatially corresponding response, while it often requires some stimulus-response translation in the Eriksen flanker task.

The effect of *a* is very similar to $\tau$, which is not too surprising as both jointly determine the time of maximum automatic activation. Thus, given a fixed value of $\tau$, this maximum is reached later, when *a* increases. The mean congruency effect decreases for small or large values of *a*. The delta function is negatively sloped for small values and positively sloped for large values of *a*. Accuracy decreases in the first RT bins with larger values of *a* in congruent trials, but increases in incongruent trials.

**Fig. 6:**
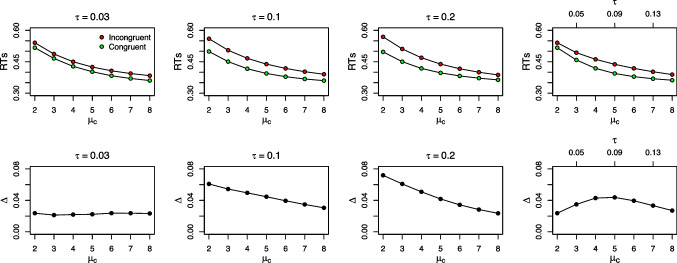
Congruency effects as a function of the controlled drift rate $\mu _c$. The *upper row* presents mean RTs in incongruent and congruent trials and the *lower row* presents the respective congruency effects $\Delta$ as a function of the controlled drift rate $\mu _c$. In the first three columns, $\tau$ takes a particular value, and the overadditivity of the congruency effect and $\mu _c$ increases the larger $\tau$. The *right-most panel* illustrates the case when $\tau$ and $\mu _c$ are correlated, which keeps the congruency effect roughly at a constant size

Why does an early peak of the automatic activation produce a negatively sloped delta function? The key idea is that, in this case, it most strongly influences early parts of the decision process. Thus, if processes terminate early (i.e., fast responses), a (large) congruency effect results. With late-terminating processes (i.e., slow responses), the influence of the automatic activation is almost gone, and hence a smaller, or even no, congruency effect results. In contrast, the later the peak of the automatic activation, the longer it influences the decision process and fast responses are less affected than slower ones.

Finally, we return to the empirical observation that congruency effects can remain of similar size when manipulating the efficiency of the controlled route (Servant et al., 2014). According to Fig. 5, a slight tendency for larger congruency effects occurs with smaller values of $\mu _c$, which arguably contrasts with such empirical results. Interestingly, already Ulrich et al. (2015) presented a simulation, and concluded that DMC accurately predicts an invariant congruency effect with varying values of $\mu _c$. The reason for these different patterns lies in the settings for $\tau$, as is visualized in Fig. 6. The left-most panels show the congruency effect for a small value of $\tau$, as was used in Ulrich et al. (2015). When the peak of the automatic process occurs early, the congruency effect indeed remains fairly stable. However, when the peak occurs later in time, the congruency effect actually becomes larger with a decreasing $\mu _c$ (see the second and third column). A similar effect has earlier been shown in Fig. 2 of Luo, Hao, Ma, and Wang (2024). To somewhat counteract this tendency, $\mu _c$ and $\tau$ need to be correlated (see right-most column).

### Summary

To summarize, DMC translates dual-route models into two independent diffusion processes: a linear diffusion process reflects controlled processing, and a pulse-like function reflects automatic processing. By varying the time point of where the latter maximum activation is reached, $t_{\text {max}}$, DMC can predict negatively and positively sloped delta functions. Hence, DMC can account for data from Simon as well as Eriksen flanker tasks, with a substantially earlier $t_{\text {max}}$ in Simon than in Eriksen flanker tasks (see already Ulrich et al., 2015, and many others). Furthermore, DMC can predict lower accuracy for the first RT bins of the CAF, particularly in incongruent trials. Allowing for assessing whether an experimental effect affects processing along the controlled or the automatic route (or both), by examining the respective parameters as a function of different experimental conditions, is a strength of DMC, and in this section, we showed that several parameters affect the mean congruency effect and the shape of the delta functions.

However, some predictions of DMC do not align perfectly with typical observations in conflict tasks. First, for some extreme parameter settings, particularly for small $\mu _c$ and *b* values, DMC predicts lower accuracy for longer RTs in congruent trials, which empirically are usually stable and high. Additionally, when $\tau$ is small (as would be the case in Simon tasks), variations of the controlled drift rate $\mu _c$ do not much affect the size of the mean congruency effect (although a small effect is indeed visible, see already Ulrich et al., 2015). Yet, when $\tau$ increases and becomes larger (as would be the case in Eriksen flanker tasks), the mean congruency effect becomes smaller the larger $\mu _c$ is. This contrasts with an empirical result reported by Servant et al. (2014; see also Stafford et al., 2011).

## Variations of DMC and other related models

The previous sections considered the standard DMC as it is typically applied to variants of traditional conflict tasks with congruent and incongruent conditions (e.g., Ellinghaus, Karlbauer, Bausenhart, & Ulrich, 2018; Hübner & Töbel, 2019; Mittelstädt et al., 2023). Of course, other models have been developed for conflict tasks, and for completeness, we briefly mention the *dual-stage two-phase model* (DSTP; Hübner et al., 2010) and the *shrinking spotlight model* (SSP; White et al., 2011; see Heuer et al., 2023; Miller & Schwarz, 2021; Wühr & Heuer, 2018, for further models for conflict tasks).

Both models explicitly describe how an individual’s attention changes over the course of a trial, but have predominantly been developed for the Eriksen flanker task, although DSTP has been extended to fit the Simon task as well (Hübner & Töbel, 2019). DSTP distinguishes a first, nonselective phase from a second phase, where processing is more selective based on the target or the flankers. During the first phase, the central process of response selection is influenced by both the target and the flankers, making the decision process particularly prone to the irrelevant flankers. Whether the target is or the flankers are the basis for the second phase depends on the outcome of another diffusion process running in parallel. A core feature of DSTP is that attention shifts are abrupt, with processing being constant within each phase, but changing instantly when moving from phase 1 to phase 2. The SSP model was proposed as an alternative to DSTP, arguing that attention in a flanker task narrows on the target in a more gradual than abrupt way. Specifically, SSP implements the zoom-lens metaphor (Eriksen & St. James, 1986), which shrinks over time thereby increasing the focus on target processing. In essence then, DSTP and SSP provide an alternative to DMC, particularly in the context of the Eriksen flanker task. A key difference between these models is that DSTP and SSP aim to precisely describe how attention chooses among the task-relevant and task-irrelevant features of a stimulus display. In contrast, DMC is a little more agnostic, focusing more closely on describing the activation pattern within the automatic and controlled route of stimulus processing. However, a more thorough discussion of these and other alternative models is beyond the scope of the present manuscript.

DMC itself has also experienced some variations for specific (experimental) situations, and these are briefly summarized next (and some are discussed in more detail in the later Section “Applications of DMC within the last 10 years”). In addition, two further models have explicitly been formulated with reference to DMC: the Revised Diffusion Model for Conflict tasks (RDMC; Lee & Sewell, 2024) and the Dual-Route Evidence Accumulation Model (DREAM; López & Pomi, 2024). Readers who are, for now, more interested in learning how and where the standard DMC has been applied since may want to skip this section and move to the subsequent section directly.

### Variations of DMC

As one example for a variation of DMC, Evans and Servant (2022) estimated the amplitude *A* separately for congruent and incongruent trials, as opposed to the standard restriction that $A_\text {incongruent} = -A_\text {congruent}$. Further, evidence accumulation according to standard DMC starts without any systematic bias, that is, with $\mathbb {E}[\textbf{X}(0)]=0$. As a second example, Luo, Yang and Wang (2023) introduced a bias parameter that shifts the starting point toward the upper or lower boundary. A third example is the Multimodal Diffusion Model for Conflict tasks (MDMC; Mahani, Bausenhart, Ahmadabadi, & Ulrich, 2019). That study investigated the simultaneous influence of an additional task-irrelevant stimulus feature in visual Simon tasks. An additional irrelevant tactile stimulus (Exp. 1) and, albeit to a lesser degree, an additional irrelevant auditory stimulus (Exp. 2), affected RTs similar to the visual stimulus location. To model the data within DMC, two automatic activations were implemented as two separate (rescaled) Gamma distribution functions that again superimpose with the controlled processing. This extension was able to fit the data reasonably well.

A final example is one of a modification of the automatic processing in the context of congruency sequence effects. These effects arise when not only the congruency of the current Trial *N*, but also that of the previous Trial $N-1$ is taken into account. Typically, congruency effects are substantially reduced following incongruent compared to congruent trials $N-1$. To model the empirical observation of a reversed Simon effect after an incongruent trial, Koob et al. (2023b) allowed the automatic activation to undershoot when returning to zero (as was already suggested by Ulrich et al., 2015). To this end, they took a difference between two pulse-like functions, with the second function reaching its maximum after the first.

These modifications were tailored to specific theoretical questions and we are not aware that they have been pursued in depth further. Also, only Evans and Servant (2022) and Luo et al. (2023) reported a parameter recovery for their particular model.^5^ Thus, we consider these modifications “custom” variants of DMC, which require more research and theoretical discussions before incorporating them into the “standard variant” of DMC.

**Fig. 7:**
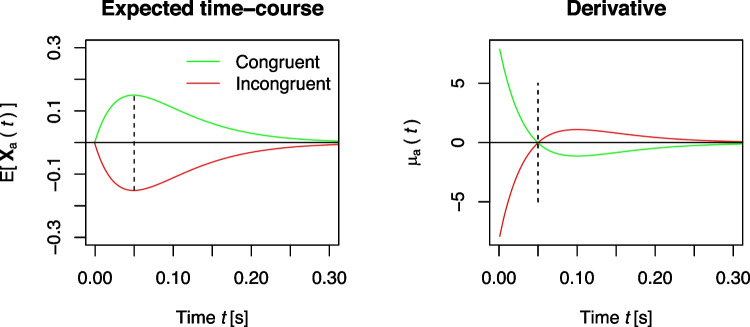
Illustration of the expected time-course within the automatic route and its derivative. The *left panel* illustrates one example of the expected activation within the automatic route, while the *right panel* illustrates the corresponding drift rates $\mu _a(t)$, that is, the first derivative with respect to time *t*

### Related models

#### The Revised Diffusion Model for Conflict tasks (RDMC; Lee & Sewell, 2024)

Lee and Sewell (2024) proposed a revised version of DMC. In their article, they argued that the assumption of a pulse-like activation function within the automatic route (i.e., the Gamma distribution function) is not plausible and therefore represents a shortcoming of DMC (see also Heuer et al., 2023, for a similar argument). We will briefly cover the two parts of this argument, which are treated in depth in a comment on RDMC (see Janczyk et al., 2025), before summarizing the architecture of RDMC itself.

First, let us take the pulse-like activation for granted for a moment, as this is DMC’s assumption of the expected time-course (see the left panel of Fig. 7). In the previous section, we mentioned that the drift rate of the automatic process, $\mu _a(t)$, is the first derivative of the (rescaled) Gamma distribution function with respect to time *t* (see Eq. 5), and examples are shown in the right panel of Fig. 7.

Clearly, the drift rate changes sign after reaching the maximum activation: In congruent trials, the drift rate starts positive, it then becomes negative, and eventually approaches zero. In incongruent trials, the drift rate starts negative and changes to positive (see the green and red lines before and after the black vertical line in Fig. 7). Lee and Sewell (2024, p. 7) suggested that this switch in sign requires “selectively accumulating ‘counter-evidence’ that is contrary to the properties of the distractor stimuli”, although the stimulus itself does not change. This notion seems to result from adopting an interpretation linking drift rate to stimulus quality (see, e.g., Ratcliff & McKoon, 2008), and that evidence for one response implies counter-evidence for the opposite response, which is quite common when drift rates are time-independent (as is the case in the standard DM or with the controlled drift rate $\mu _c$ in DMC). In the case of the standard DM, fitting two separate drift rates to stimulus conditions requiring different responses results in drift rates of different signs. Whether and how this interpretation makes sense with time-dependent drift rates is open, though.

Second, the general form of the expected automatic activation implies that activation fades after reaching its peak, either due to decay or inhibition. Lee and Sewell (2024) argued, however, that activation, once gathered, should remain in the system and question “that inhibition can reverse or undo the effects of previously completed processing” (p. 6). We do not discuss the plausibility of inhibition in this review, but, several fields of psychology, where inhibition is indeed conceived as capable of reducing activation, are presented in Janczyk et al. (2025).

To avoid decreasing activation, while still being able to predict both positively and negatively sloped delta functions, Lee and Sewell (2024) presented RDMC. This model is also a dual-route model. However, both routes continuously provide output, although their relative weight changes with increasing time *t*. The weight of the automatic route, $w_a(t)$, decreases over time, while the weight of the controlled route, $w_c(t)$, increases reciprocally. Specifically, the corresponding weights are defined as

7 $$\begin{aligned} w_a(t) = A_0 e^{-k t} \quad \text {and}\quad w_c(t) = 1 - w_a(t)\,, \end{aligned}$$

where $A_0 \in [0;1]$ is the automatic route’s weight at time $t=0$ and *k* is the decay rate. Multiplication of these weights with the base drift rates of the automatic and the controlled route, $d_a$ and $d_c$, yields the (time-dependent) drift rates of both routes. These are finally added to derive the overall drift rate

$$ \mu (t) = w_a(t)d_a+w_c(t)d_c\,. $$

An important point is that RDMC assumes separate decay parameters $k_c$ and $k_i$ for congruent and incongruent trials, respectively. Only then can RDMC predict negatively sloped delta functions. The assumption of different decay parameters requires the cognitive system to “know” the congruency of a trial already at the beginning of response selection (i.e., of the diffusion process). The authors suggest that congruency is “a high-order emergent feature of the stimulus configuration that is based on the homogeneity of stimulus elements (e.g., as in Eriksen flanker tasks) or the conceptual alignment of elements (e.g., as in Stroop and Simon tasks)” (Lee & Sewell, 2024, p. 9). This is conceivable for some variants of conflict tasks, like the Eriksen flanker task. However, a standard Simon task is one counterexample. In fact, knowledge about the required response seems necessary to determine congruency in that case, and this knowledge is not available at the beginning of the diffusion process.

A final point worth mentioning concerns the parameter recovery properties of RDMC. While Lee and Sewell (2024) reported good properties, Janczyk et al. (2025) argued that their procedure was not ideal. In more detail, parameter estimation was done with the starting points being selected from the vicinity of the generating parameters. Clearly, when applying RDMC, we would not know the true generating parameters beforehand. When using a minimization algorithm with a larger parameter space, recovery was not good for the parameters $A_0$ and $k_c$, even for large trial numbers (see also Appendix A in Lee, Ballard, & Sewell, 2025a).

In sum, RDMC is a valuable attempt to handle both positively and negatively sloped delta functions, and it can fit data from both Simon and Eriksen flanker tasks very well (Janczyk et al., 2025; Lee et al., 2025a; Lee & Sewell, 2024). In addition, RDMC does not require the output (i.e., derivative) of the automatic process to switch sign, which has been proposed as a conceptual limitation of DMC (Heuer et al., 2023; Lee & Sewell, 2024). While all arguments and counterarguments are certainly subject to discussion and a matter of perspective, the currently less-than-optimal recovery for some parameters undermines the utility of RDMC. Future research should explore whether variants of RDMC can improve parameter recovery.

#### Dual-Route Evidence Accumulation Model (DREAM; López & Pomi, 2024)

Another potential drawback of DMC was raised by López and Pomi (2024). These authors argued that DMC can only be seen as an “*ad hoc* approximation to the time course of activation of the decision-making process for conflict tasks” (p. 1509). Preferably, results obtained with DMC can be reproduced “with more relaxed assumptions, ideally with implicit time dependencies that emerge from interactive dynamics” (p. 1509). In other words, the Gamma distribution function used in DMC is an inherent assumption of the model; it is specified *as is*, with the rate $\mu _a(t)$ of automatic activation explicitly depending on *t*. As a result, DMC cannot be regarded as an autonomous system that changes dynamically with respect to the activation within the automatic and controlled route.

To elaborate on this issue, the authors explicitly implemented inhibition (Ridderinkhof, 2002a) into their model: The more the controlled route becomes activated, the more the activation in the automatic route becomes suppressed. As in DMC, the controlled and the automatic route’s activation are conceived as separate diffusion processes that are eventually added to yield the net activation of the overall decision process. Both processes comprise their own drift rate, $\mu _c$ and $\mu _a$, and the two processes can be written as the difference equations

8 $$\begin{aligned} \mathbf {X_c}(t+1)&= \mathbf {X_c}(t)+\mu _c+\textbf{Z}\sigma _c \end{aligned}$$

9 $$\begin{aligned} \text {and } \mathbf {X_a}(t+1)&= \mathbf {X_a}(t)+\mu _a\boxed {-\gamma |\mathbf {X_c}(t)| \mathbf {X_a}(t)}+\textbf{Z}\sigma _a \,. \end{aligned}$$

Here, $\textbf{Z}$ is the standard normal distribution, and $\sigma _a$, and $\sigma _c$ are diffusion constants for each (sub)process.^6^ Equation 8 represents the evidence accumulation within the controlled route, $\mathbf {X_c}(t)$, and describes a standard diffusion process with a time-independent drift rate $\mu _c$. That is, for each time step *t*, the evidence value at the next step, $\mathbf {X_c}(t+1)$, is derived from the current evidence value, $\mathbf {X_c}(t)$, plus the drift rate and random noise. Setting aside the boxed part, Equation 9 is equivalent to Equation 8, only that the drift rate is now $\mu _a$, as it represents the evidence accumulation within the automatic route. The crucial point is the boxed part, which consists of several factors: $\mathbf {X_c}(t)$ is the current activation within the controlled route, which is subtracted from activation within the automatic route after being scaled with the inhibition parameter $\gamma$ (with $\gamma > 0$). This suppression, however, also depends on the current activation within the automatic route, $\mathbf {X_a}(t)$. First, this is necessary to prevent the entire product from taking values below zero, which would ultimately force the automatic route’s activation to become negative. This also makes sense psychologically, because, if no or only little activation is present in the automatic route, that is, if $\mathbf {X_a}(t)$ approaches zero, then not much inhibition can be exerted. Second, including $\mathbf {X_a}(t)$ in the product also ensures that an early and rapid increase in the automatic route’s activation is accompanied by rapid suppression. This is the requirement for the model to predict negatively sloped delta functions.

Indeed, DREAM yields shapes of the automatic activation that are similar to what DMC assumes. If the drift rate of automatic processing $\mu _a$ and the inhibition parameter $\gamma$ are relatively small, a late peak of automatic activation emerges, resulting in positively sloped delta functions. If $\mu _a$ and $\gamma$ are relatively large, in contrast, an early peak emerges, resulting in negatively sloped delta functions (for illustrations, please see Figs. 1 and 2 in López & Pomi, 2024). When being fitted to empirical data, López and Pomi conclude that “DREAM does not provide qualitative or quantitative advantages over DMC” (p. 1519). Yet, the work clearly shows that the assumed shape of the automatic activation within DMC is reasonable from an inhibition stance and can emerge from the model dynamics itself, rather than from an explicit implementation as in DMC. To date, an extensive parameter recovery study for DREAM is lacking though.^7^

#### Summary

Two models have recently been proposed with direct reference to DMC (Lee & Sewell, 2024; López & Pomi, 2024). RDMC’s critique targets the shape of the automatic activation and its resulting drift rate, which changes sign during the trial. As an alternative, RDMC uses time-varying weights that scale the constant output from the controlled and automatic route. Yet, to date, some of RDMC’s parameters cannot be recovered adequately. DREAM explicitly implements suppression of the automatic activation, as envisaged by Ridderinkhof (2002a), and offers a reasonable account for the shape of the automatic function, which emerges from the model dynamics alone. In sum, RDMC and DREAM appear as more oriented toward specific processes that are at play in conflict tasks, but come with some problems, such as parameter recovery. We continue with our focus on DMC and turn toward its applications over the past decade.

## Applications of DMC within the last 10 years

DMC has been applied to a variety of tasks and phenomena since its introduction. We begin by providing a brief (and likely non-exhaustive) overview of the standard tasks used in cognitive psychological research. More space is then devoted in the subsequent sections to particular empirical phenomena and modifications of the standard DMC. This section is most relevant for readers interested in how DMC can be used, what research questions have been tackled, and in what ways DMC has proven to be a useful tool. Those more interested in technical aspects and developments of DMC, may want to skip this section and move to the next one, where such topics are covered.

### Beyond Simon and Eriksen flanker tasks

It seems fair to say that DMC has been primarily and most successfully used to model data from Simon and Eriksen flanker experiments (among them Ellinghaus et al., 2018; Ellinghaus, Liepelt, Mackenzie, & Mittelstädt, 2024a, b; Evans & Servant, 2020, 2022; Hübner & Töbel, 2019; Janczyk et al., 2025; Kelber, Mittelstädt, & Ulrich, 2025a; Koob et al., 2023b; Mittelstädt et al., 2023; Servant & Evans, 2020; Servant et al., 2016; Ulrich et al., 2015). Less prominent is its application to the Stroop task (Stroop, 1935), even though this task is often mentioned when introducing conflict tasks (see also our Fig. 1). To the best of our knowledge, data from a vocal color-naming Stroop task have only been analyzed with DMC in Appendix B of Kelber, Ulrich, Mackenzie, Jeschke, and Mittelstädt (2025b). Because the underlying data (Spinelli & Lupker, 2023) were collected with an additional manipulation of the proportion of congruent trials, we discuss this study below.

Few available studies fitted DMC to a manual Stroop task. A comparative analysis of Simon, Eriksen flanker, and (manual) Stroop tasks was presented by Hedge, Powell, Bompas and Sumner (2022, Table C1), based on their own data and the data of Hedge, Powell, Bompas, and Sumner (2018a), Hedge, Vivian-Griffiths, Powell, Bompas, and Sumner (2019), and Whitehead, Brewer, and Blais (2019). The overall conclusion from this comparison is that the time-to-peak parameter “followed the expected pattern of being shortest for the Simon tasks and longest for the Stroop” task (Hedge et al., 2022, p. 1454). Roughly, the peak typically occurs earlier than 100 ms for Simon tasks, between 100 and 150 ms for Eriksen flanker tasks, but much later for Stroop tasks (even around or later than 500 ms).

Some studies employed variants of Stroop-like tasks. Kelber et al. (2025b) used a *counting Stroop task* where the number words ONE, TWO, THREE, and FOUR (or the corresponding Arabic numerals) were presented in 1, 2, 3, or 4 rows one below the other (see Bush et al., 1998). Participants were to manually indicate the number of rows, and to ignore the words/numerals presented. This study is considered below in more detail, as it involved a proportion congruency manipulation as well. Further, the study by Whitehead et al. (2019) used a *spatial Stroop task* (which they called a Simon task, but see their Footnote 3), where the words UP, DOWN, LEFT, and RIGHT appeared above, below, left of, and right of the screen center. Participants were to press the arrow key corresponding to the word meaning (and to ignore the location). Regardless of the exact variant, the estimated time of maximum automatic activation was relatively late.

An overall similar pattern across the Simon, Eriksen flanker, and Stroop task has been reported for children by Ambrosi, Servant, Blaye, and Burle (2019), though with different (absolute) time-courses. In that study, a standard Simon task, a child-friendly Eriksen flanker task, and a child-friendly *color–object Stroop task* (adapted from Archibald & Kerns, 1999) were used. In the Simon task, stimuli were blue and red circles presented to the left or right of the screen center. The children responded to the color with a left or right key press, while ignoring the circle position. In the Eriksen flanker task, a central fish swimming either to the left or right was presented, flanked by four fish swimming either in the same or opposite direction. The task was to indicate the swimming direction of the central fish. Finally, in the color-object Stroop task, line drawings of a salad and a carrot were presented in green or orange color, but the (manual) response was determined by the canonical color of the object (green for salad, orange for carrot). Matching with other studies (e.g., Hedge et al., 2022), the activation in the automatic route reached its maximum early in the Simon task (i.e., $\tau = 59.4$), and substantially later in the Eriksen flanker and Stroop task ($\tau = 441.8$ and $\tau = 504.2$, respectively). In other words, automatic activation built up and declined fast relative to the activation in the controlled process in the Simon task, while this took more time in the other two tasks. Despite these similarities, the results for the Eriksen flanker task are particularly interesting, because the corresponding maximum of the automatic activation occurred substantially later than in studies with adults (e.g., Ulrich et al., 2015), and only slightly below the estimate for the color-object Stroop task. Second, the CAFs showed fast errors also for the congruent condition.

### Neutral trials in conflict tasks

So far, we have only focused on congruent and incongruent trials in conflict tasks. It is still unclear whether the congruency effect results from irrelevant activation speeding responses in congruent trials (“facilitation”), hindering responses in incongruent trials (“interference”), or both, possibly to varying degrees. Mathematical models of conflict tasks can help clarify this, and from a DMC perspective, it is evident that both facilitation and interference contribute to the congruency effect.

The empirical assessment of these contributions requires including a neutral (baseline) condition (see, e.g., Besner, Stolz, & Boutilier, 1997; Jonides & Mack, 1984; MacLeod, 1991; Posner & Snyder, 1975), where “the stimulus should contain irrelevant information that does not promote either of the two response alternatives or prompts both responses to the same degree” (Smith & Ulrich, 2024, p. 2026). However, in practice, identifying appropriate neutral conditions is challenging, and empirical results show significant variability. Smith and Ulrich suggested that, if neutral conditions correspond to the mid-point of congruent and incongruent conditions, the equation

$$ RT_\text {neutral}-RT_\text {congruent} = RT_\text {incongruent}-RT_\text {neutral} $$

should hold, or in other words, the ratio

$$ R=\frac{RT_\text {neutral}-RT_\text {congruent}}{ RT_\text {incongruent}-RT_\text {neutral}} $$

should approximately be equal to 1. Values of $R>1$ indicate stronger facilitation than interference effects, while $R<1$ indicates the opposite. However, an empirical review of multiple studies shows that $R=1$ is rarely observed (see Table 1 in Smith & Ulrich, 2024). For Eriksen flanker and Stroop tasks, $R<1$ is observed most often (meaning smaller facilitation than interference effects). For the Simon task, the picture is more heterogeneous. While in the majority of studies $R<1$ occurred, the number of studies showing $R>1$ (meaning larger facilitation than interference effects) was higher for the Simon than for the Stroop or the Eriksen flanker tasks. Smith and Ulrich (2024) contributed to this empirically by conducting a Simon, Eriksen flanker, and Stroop experiment, each using either linguistic or symbolic stimuli. For the Eriksen flanker task, $R<1$ was observed for both stimulus types. For the Stroop task, $R<1$ was observed for the linguistic stimuli, and $R>1$ for the symbolic stimuli. For the Simon task, $R>1$ was observed for both stimulus types (see also Mahani et al., 2019, for Simon experiments with irrelevant features from two modalities). In essence, behavioral experiments investigating the roles of facilitation and interference using neutral trials paint a rather heterogeneous picture, although there seems to be a trend for smaller facilitation than interference effects ($R < 1$), particularly for the Stroop and Eriksen flanker task.

How can DMC help identify the existence and contributions of facilitation and interference? DMC, as initially proposed, assumes similar activation within the automatic route in both congruent and incongruent conditions, consistent with dual-route model assumptions (Kornblum et al., 1990). In other words, the (absolute) amount of information contributed by the automatic process is always the same, irrespective of the congruency of a trial. However, two extensions of DMC have been suggested to specifically address neutral conditions and to distinguish facilitation from interference.

First, Evans and Servant (2022) suggested that “it seems plausible that [...] facilitation and interference could be asymmetric” (p. 1189). Accordingly, they extended DMC by assuming different amplitudes, $A_\text {congruent}$ and $A_\text {incongruent}$ for the congruent and incongruent conditions, respectively. This DMC extension was fitted to (published) data from three Simon and six Eriksen flanker experiments. The general conclusion was that for a large number of participants, only interference was visible, that is, the estimate of $A_\text {congruent}$ was close to zero and much smaller in magnitude than that of $A_\text {incongruent}$. If anything, this effect was more pronounced in the Eriksen flanker task than in the Simon task, although it remained the primary finding in both cases.

Second, Smith and Ulrich (2024) pointed out that the original DMC does indeed violate the $R=1$ assumption. In fact, for many parameter constellations, DMC predicts $R>1$, that is, a larger contribution of facilitation than of interference. Only for some parameter combinations, DMC tends to make the opposite prediction. One such case is when RTs are relatively short due to a low boundary setting (as, e.g., a result of speed instructions; Smith & Ulrich, 2025). As in their literature overview $R<1$ occurred predominantly, the authors concluded that DMC, in its original form, cannot accurately describe the contribution of facilitation and interference in conflict tasks. This nicely aligns with the modeling results by Evans and Servant (2022), where $A_\text {congruent}$ turned out smaller than $A_\text {incongruent}$. Obviously, if the amplitude is smaller in congruent than in incongruent trials, DMC predicts $R<1$. However, Smith and Ulrich discussed another way of modeling $R<1$ without relaxing the assumption of an equally strong amplitude of the automatic process. Specifically, they suggested time-dependent controlled processing. For example, the drift rate of the controlled process might follow an exponential decay function,

$$ \mu _c(t)=\mu _c e^{-ct}\,, $$

so that the corresponding expected time-course of controlled processing is concave. Note that, with $c\rightarrow 0$, $\mu _c(t)$ becomes more and more constant and thus the original DMC emerges as a special case. The authors speculated that, with this change, DMC can predict a range of *R* values and handle asymmetric facilitation and interference effects. Importantly, however, the authors did not present model simulations or model fits. To fill this gap, we ran the proposed DMC variant with typical DMC values for the Eriksen flanker and Simon task, and present the results in Electronic Supplement C. We could not demonstrate that a concave controlled process easily predicts $R < 1$, however. Rather, the standard DMC and the DMC with a concave controlled process predicted similar *R* values. However, the simulations were far from exhaustive. Thus, it might still be that a concave controlled process predicts $R < 1$ for some parameter constellations, but it does not seem to predict $R<1$ for the ones we have used. A more thorough treatment of the suggestions by Smith and Ulrich (2024) is warranted.

In sum, a model-based approach can certainly be a helpful tool to separate facilitation and interference in the overall congruency effect. At the same time, this remains a field with potential, as relatively few studies have explicitly applied DMC to this topic, and both the modeling approaches and the empirical findings are inconsistent.

### Local adaptations: Sequential modulations and the congruency sequence effect

A particularly reliable observation for congruency effects in conflict tasks is their sequential modulation. This means that the congruency effect (based on the congruency in Trial *N*) depends on the congruency of the previous Trial $N-1$: Congruency effects are larger following congruent than following incongruent trials, after which they are sometimes even reversed (in particular for the Simon task). This *congruency sequence effect* was first reported by Gratton, Coles, and Donchin (1992) and has been replicated many times for the various conflict tasks (for reviews, please see Braem, Abrahamse, Duthoo, & Notebaert, 2014; Duthoo, Abrahamse, Braem, Boehler, & Notebaert, 2014; Egner, 2007; Schmidt, 2019).

One important class of explanations for the congruency sequence effect assumes that the influence of the task-irrelevant feature is reduced as a consequence of just experienced conflict. Hence, the congruency effect becomes smaller on the subsequent trial. In contrast, following a congruent trial, the influence of the irrelevant feature is greater, as it did not previously hinder performance. Attributed initially to different expectancies resulting from an incongruent or congruent trial, later accounts placed more emphasis on learning (Verguts & Notebaert, 2008) and conflict-induced attentional shifts. Arguably, the most frequently referenced account is the conflict-monitoring theory (Botvinick, Braver, Barch, Carter, & Cohen, 2001): Conflict, operationalized as the simultaneous activation of two responses, is detected by a conflict monitor. This signal is then used to adjust processing for the upcoming trial.

Alternatively, it has been suggested that the congruency sequence effect results from feature integration and a particular constellation in many conflict tasks (Hommel, Proctor, & Vu, 2004; Mayr, Awh, & Laurey, 2003). More precisely, congruent-congruent (cC) and incongruent-incongruent (iI) transitions from Trial $N-1$ to Trial *N* are often full repetitions or full alternations of stimulus-response episodes, while incongruent-congruent (iC) and congruent-incongruent (cI) transitions are often partial repetitions. As the latter episodes lead to longer RTs, the pattern of a congruency sequence effect becomes visible without any conflict-related adaptation. To date, the fairest conclusion seems to be that both aspects contribute to the congruency sequence effect (Lee, Verhaeghen, Hazeltine, & Schumacher, 2025b).

Assuming that conflict adaptation is (at least) part of the congruency sequence effect, it is of interest what aspects of task-processing are affected in the course of conflict adaptation: processing along the controlled route, along the automatic route, or both? Bausenhart, Ulrich, and Miller (2021, p. 825) considered DMC explicitly in their discussion and suggested that “behavioral adaptation in conflict tasks may be modeled through changes in the amplitude of the automatic activation peak (Ulrich et al., 2015). For instance, the automatic activation peak may be suppressed after a recent or frequent experience of conflict.” To date, two studies used DMC to investigate this question and provide a larger background on this topic (Koob et al., 2023b; Luo & Proctor, 2022).^8^

Luo and Proctor (2022) extended DMC in a way that specific parameter constellations were used for the four possible congruency transitions. More precisely, they argued that (1) feature integration affects controlled processing, and hence $\mu _c$, and that (2) experiencing an incongruent trial affects automatic processing, and hence $\mu _a(t)$:

1. Controlled processing is assumed to be more efficient in trials with complete repetitions or complete alternations (iI, cC) than in those with partial repetitions (iC, cI). Hence, a drift rate $\mu _{cL}$ was implemented for the former trials, and a drift rate $\mu _c$ (with $\mu _c<\mu _{cL}$) was implemented for the latter trials.
2. Similarly, trials following an incongruent trial (iI, iC) are assumed to employ the automatic drift rate $\mu _{aS}(t)$ while trials following a congruent trial (cI, cC) employ the drift rate $\mu _a(t)$. Both drift rates differ in the parameters *A* and $\tau$ (with $a=2$).

Table 2 summarizes the drift rates of the superimposed diffusion processes according to Luo and Proctor (2022, see also their Table 1).^9^ This re-parameterized DMC, which they termed CSE-DMC, was fitted to data from three different experiments, of which the first study was a standard visual horizontal Simon task with color stimuli. The performance of CSE-DMC was additionally compared to two other models, each only incorporating either the (feature-integration) influence on controlled processing or the (conflict-related) influence on automatic processing. Generally, CSE-DMC outperformed these two model versions and accounted well for the congruency sequence effect, that is, for the delta functions following congruent and incongruent trials. Regarding parameter estimates, *A* and $\tau$ were estimated slightly smaller for the iC and the iI transitions compared with the cC and the cI transitions (see Table 2 in Luo & Proctor, 2022). This suggests that experiencing conflict in an incongruent trial results in an earlier, less pronounced maximum of automatic activation in the following trial.

**Table 2:** Summary of CSE-DMC’s drift rates

Trial sequence	Superimposed drift rate $\mu (t)$
cC	$\mu _{cL} + \mu _a(t)$
cI	$\mu _{c} - \mu _a(t)$
iC	$\mu _{c} + \mu _{aS}(t)$
iI	$\mu _{cL} - \mu _{aS}(t)$

Trial sequence identifies the transition of the congruency level, with the first letter indicating congruency in the previous Trial $N-1$, and the second (capital) letter the congruency of the current Trial *N*

The second study, using DMC to investigate the congruency sequence effect, was conducted by Koob et al. (2023b). In a first approach, DMC was fitted to six published datasets from Simon and Eriksen flanker experiments. The congruency sequence effect was observable in all these data sets and, notably, the Simon effect was slightly reversed following incongruent trials. A reversal of the congruency effect is not possible with the standard version of DMC, however, as the automatic activation declines to zero, but does not “under- or overshoot” (in congruent and incongruent conditions, respectively). Hence, while the standard DMC was used to fit the Eriksen flanker data, a modified version of DMC was used for the Simon data. The approach to this was to model the expected time-course of the automatic activation as the difference of two Gamma distribution functions, that is, as (using the notation of the original article)

$$\begin{aligned} \mathbb {E}[\mathbf {X_a}(t)]&= \Gamma _\text {diff}=\Gamma (t|A_1, \tau _1, a_1) - \Gamma (t|A_2, \tau _2, a_2) \\&=\mathbb {E}[\mathbf {X_{a,1}}(t)] - \mathbb {E}[\mathbf {X_{a,2}}(t)]\,, \end{aligned}$$

where $\Gamma$ is used to denote the Gamma distribution function of DMC (see Eq. 4), thus

$$\begin{aligned} \mathbb {E}[\mathbf {X_a}(t)]&= \left( A_1 e^{-\frac{t}{\tau _1}}\cdot \left[ \frac{t e}{(a_1-1)\tau _1}\right] ^{a_1-1} \right) \\&\quad -\left( A_2 e^{-\frac{t}{\tau _2}}\cdot \left[ \frac{t e}{(a_2-1)\tau _2}\right] ^{a_2-1} \right) \,. \end{aligned}$$

To (somewhat arbitrarily) reduce the model complexity, $a_1=a_2=2$ and $\tau _2=2.5 \tau _1$ were implemented as restrictions. Feature integration was accounted for in additional models by allowing the controlled drift rate $\mu _c$ (as in Luo & Proctor, 2022) and the non-decision time *t*0 to vary for cC/iI versus cI/iC congruency transitions. The major result from these analyses is that experiencing an incongruent trial primarily reduces the amplitude *A* of activation within the automatic route. The addition of parameters capturing the confounds did not change the conclusion.

A second approach of Koob et al. (2023b) relied on changing the parameters dynamically as a result of experiencing conflict (or not) in a simulation. In each trial, conflict was quantified as the maximum difference in activation for the correct and incorrect response. This value was then mapped to the parameters $\mu _c$, *A*, and/or $\tau$, which were adjusted before the next trial’s simulation started. Different (combinations of) parameters were tested as the target of this adjustment. The overall conclusion from these simulations aligned with the model fitting results: Only reducing the amplitude *A* after incongruent trials yielded the typical congruency sequence effect.

For completeness, a recent study on the congruency sequence effect was conducted with RDMC (Lee et al., 2025a). In this study, Simon and Eriksen flanker data were used, both in a standard version and in a version that avoided stimulus and response repetitions. The authors found that congruency sequence effects in the Simon task are primarily driven by top-down adjustments in automatic processing, matching with the results summarized above. For the Eriksen flanker task, results did not clearly support a conclusion regarding controlled or automatic processing.

In sum, analyses with DMC (and RDMC) suggest that the congruency sequence effect arises from a reduced amplitude (perhaps in combination with an earlier peak) of the automatic activation. A similar conclusion has recently been advanced by Gheza and Kool (2025) who used a multidimensional task where 0–3 features of a current stimulus could be congruent with the target. Data from this task were modeled with a neural network and a subsequent diffusion process. Sequential adaptations were attributed to the suppression of irrelevant features. However, the results also showed that each potential conflict dimension was monitored independently of the others, and conflict was detected on a level different from conflicting overall response activations (such as in Koob et al., 2023b).

### Global adaptations: Proportion congruency effects

In most studies, congruent and incongruent conditions appear in 50% of the trials each. This is not necessary, of course, and already Gratton et al. (1992) varied the proportion of congruent trials in an Eriksen flanker task between 75%, 50%, and 25% of the trials. Often, and as well in the following, the conditions with 25% and 75% congruent trials are referred to as *mostly incongruent* and *mostly congruent* conditions, respectively. As a result, the flanker congruency effect was larger, the higher the percentage of congruent trials (i.e., larger in mostly congruent than in mostly incongruent conditions). This observation is known as the *(listwide) proportion congruency effect* and has been replicated across other tasks (for a review, see Bugg & Crump, 2012). The Simon effect is often even reversed in conditions with a large proportion of incongruent trials (e.g., Hommel, 1994; Marble & Proctor, 2000; Ridderinkhof, 2002b).

Remember that one explanation for the congruency sequence effect assumes that the cognitive system adapts its processing at the local level according to the conflict experienced in the just preceding Trial $N-1$. Similarly, one explanation for the proportion congruency effect assumes *proactive control*, that is, the cognitive system strategically adjusts relevant and/or irrelevant processing according to the overall experience of conflict on a global level (Botvinick et al., 2001; Bugg & Crump, 2012).

Luo et al. (2023) investigated whether the proportion congruency effect and the reversal of the Simon effect are the result of learned links between the task-irrelevant stimulus feature and a corresponding response. Their argument is basically that biases toward one or the other response exist in the different proportion congruency conditions and whether the trial is congruent or incongruent. If the majority of trials are incongruent, a left stimulus should bias the decision process toward a right response and a right stimulus should bias it toward a left response. These responses would be correct in incongruent trials, but incorrect in congruent trials. In DMs, such biases have been implemented by introducing a corresponding bias parameter that systematically shifts the starting point of the decision process, $\textbf{X}(0)$, toward one of the boundaries (e.g., Ratcliff, 1978; Ratcliff et al., 2016; Voss et al., 2004).

Delta functions (reported by Ridderinkhof, 2002b, and replicated in an experiment of Luo et al., 2023) were first positively and then negatively sloped in the mostly congruent condition, while they were (only) negatively sloped and became reversed with longer RTs in the mostly incongruent condition. According to simulations and model fitting, Luo et al. concluded that this pattern could only be predicted by DMC, including a biased starting point that differs between congruent and incongruent trials in sign, but not in the absolute value. While this extension and application certainly have potential for future research, there is one possible disadvantage of this approach. As discussed by the authors already, the bias concerns a parameter that must be effective at the very beginning of the decision process. In other words, the system must know the congruency of the current trial at $t=0$ of the diffusion process proper. Note that this is not the time point of stimulus presentation, but only after the encoding part of the non-decision time has finished already. In principle, some features of the stimulus display may thus be available to the cognitive system already. Luo et al. argue though, that the system does not necessarily need this information, but only information about the spatial location, which then leads to a bias toward one of the two boundaries (see also Janczyk et al., 2025, for more discussion about a related point in the context of RDMC).

Kelber et al. (2025b) argued that the study by Luo et al. (2023) suffers from the shortcoming that the analyzed mostly congruent and mostly incongruent conditions are confounded with other possible explanatory mechanisms, such as, for example, contingency learning. To this end, they employed a manual counting Stroop task with the mostly congruent versus mostly incongruent manipulation being implemented in inducer items, while focusing on its effect on diagnostic items with the same proportion of congruent and incongruent items (see Braem et al., 2019, for more information). In two out of three experiments, Kelber et al. (2025b) observed a proportion congruency effect even in these confound-free diagnostic items (see also Spinelli & Lupker, 2023). Going further, the delta functions were positively sloped, and the slope itself was equal for the diagnostic items in the mostly congruent and mostly incongruent lists. This observation was further scrutinized by applying DMC to the data. Across the three experiments, the amplitude *A* was smaller in mostly incongruent than in mostly congruent conditions, pointing to the importance of (the strength) of automatic processing for the proportion congruency effect. A reanalysis of the data in Spinelli and Lupker (2023) with a vocal color–word Stroop task mainly yielded the same conclusions (see Appendices A and B in Kelber et al., 2025b).^10^

### Individual differences research

On the group level, congruency effects are stable, and arguably, there are few cases where an (adequately powered) experiment would fail to replicate a congruency effect. In practice, Stroop, Simon, and Eriksen flanker tasks are standard tools to assess response inhibition (e.g., Miyake et al., 2000). Theoretically, suppose this represents a latent ability measured via all these tasks (and others, such as the antisaccade and the stop-signal task). In that case, it is usually assumed that the respective congruency effects are highly correlated across individuals. Empirically, though, this is often not the case (e.g., Aichert et al., 2012; Friedman & Miyake, 2004; Hedge et al., 2018a, 2022; Rey-Mermet, Gade, Souza, Von Bastian, & Oberauer, 2019). This state has led authors to pessimistic views on whether a psychometric construct such as “inhibition” makes sense at all (e.g., Rey-Mermet, Gade, & Oberauer, 2018). However, it should be kept in mind that the congruency effect is a measure of difference. The reliability of such measures is known to be low, and therefore, their correlation with other measures must also be low (see Miller & Ulrich, 2013, for a psychometric analysis of this correlation issue). This can lead researchers to draw false conclusions that congruency effects across tasks do not share common mechanisms.

Yet, individual congruency effects in RTs and error rates may rarely result from a single underlying process. Rather, they may be additionally affected by individual differences in, for example, strategy, while typically the “resulting cost score is supposed to be immune from contamination” (Hedge et al., 2018a, p. 1201). Hedge et al. considered this from the perspective of cognitive models and explored possible correlations between RT and error rate congruency effects when several model parameters were varied in a simulation. In the case of DMC, the size of simulated congruency effects was varied via the amplitude *A*, and different individual strategies were implemented via the boundary *b*. The important point from these simulations is that, independent of the underlying model, diverse correlations between the congruency effects in RTs and error rates can arise. Noteworthy, when both amplitude *A* and boundary *b* varied, small-to-zero correlations result. This suggests that congruency effects as such are not a pure measure of the assumed underlying processing ability. They may indeed be somewhat confounded and blurred by other influences, such as strategic differences.

This view was recently supported by another study using DMC. Hedge et al. (2022) first fitted DMC to data from various conflict tasks and then analyzed correlations between DMC’s parameters across these tasks. While boundary *b* and non-decision time *t*0 exhibited correlations slightly higher than 0.5, and the drift rate $\mu _c$ at least around 0.32, those for the amplitude *A* and $\tau$ were only 0.04 on average. The second approach taken by Hedge et al. is even more telling. Here, data were simulated for a hypothetical Simon, Eriksen flanker, and Stroop task under the assumption of an underlying latent processing ability via constraining the amplitude *A* and $\tau$ to correlate with $r\in \{0.3,0.5,0.7\}$ across tasks. Despite this, the correlations for congruency effects measured in RTs or error rates were non-existent to moderate, though the absolute size seemed to depend on the task as well. In contrast, implementing correlations between drift rate $\mu _c$ and the boundary *b* yielded larger correlations between congruency effects, which were even larger for error rates than for RTs.

The results summarized above emphasize that it seems complicated to arrive at safe conclusions about underlying processing abilities in conflict tasks based on correlations between congruency effects in RTs and error rates. Indeed, this is a field of ongoing research activity (Rey-Mermet, Singmann, & Oberauer, 2025; Rouder, Kumar, & Haaf, 2023), but showcases how a modeling approach could be informative and insightful. A possibly promising avenue could be the approach suggested by Robinson and Steyvers (2023). These authors used (simplified) models of task switching and an Eriksen flanker task and tested whether certain parameters related to cognitive control can be swapped between both tasks. They concluded that “the results are consistent with the view that the flanker and task-switching tasks probe common latent cognitive control processes” (p. 84).

Future research may also want to address two other fields of importance in the realm of individual differences. First, to the best of our knowledge, only one study reported test-retest reliabilities for DMC’s parameters (Hedge et al., 2019). While for several parameters the resultant values were around 0.5, in particular $\tau$ suffers from no-to-small reliability in that study (see, e.g., Lerche & Voss, 2017; Schubert et al., 2016, for reliability estimates for the standard DM). Second, research into how DMC’s parameters are linked to measures of personality is just in the beginning. Hedge, Powell, Bompas, and Sumner (2020), for example, assessed self-reported impulsivity and reported absent correlations concerning the boundary parameter *b*, which is, however, often interpreted as reflecting different response styles.

### Aging research

A well-known result from the literature on cognitive aging is longer RTs in older compared with younger adults, often attributed to a general slowdown of cognitive processing (e.g., Salthouse, 1996; Verhaeghen & Salthouse,^1997^). DMs have for a long time been used to scrutinize the underlying reasons in more detail and (more or less) have consistently shown the non-decision time *t*0 and the boundary *b* to increase with age (e.g., Ratcliff, Spieler, & Mckoon, 2000; Ratcliff, Thapar, & McKoon, 2001; von Krause, Radev, & Voss, 2022). Particularly, the latter result points to a more conservative response style in older adults.

Regarding conflict tasks, several studies have reported increasing congruency effects with age. However, the exact developmental trajectory over the lifespan appears to vary across tasks (see Kelber et al., 2025a, for a recent summary and references). Servant and Evans (2020) were the first to use DMC to assess the effects of aging on cognitive processing. In that study, DMC (and SSP by White et al., 2011) were fitted to Eriksen flanker task data from 20 younger and older ($>60$ years) adults. Again, differences in the non-decision time *t*0 and boundary *b* were observed, while the drift rate of the controlled process, $\mu _c$, and the peak latency of the automatic process also increased with age. These results suggest a potential additional role for more efficient controlled processing and longer-lasting automatic processing in older adults.

Recently, Kelber et al. (2025a) analyzed a large dataset of 1800 participants from 21–80 years of age. These participants provided their data via an online game with a flanker-like task. As this game was repeatedly administered, practice effects were assessed simultaneously. The analyses of DMC parameters first replicated the increasing boundary *b* and longer non-decision time *t*0 with increasing age. Contrary to the results of Servant and Evans (2020), however, the drift rate $\mu _c$ decreased with age, although it increased overall with practice. The amplitude *A* showed an initial practice-related increase, but remained rather stable with age and further practice. However, similar to the results of Servant and Evans (2020), the peak of the automatic process occurred later, that is, the parameter $\tau$ was estimated to be larger with increasing age.

In sum, the studies by Servant and Evans (2020) and Kelber et al. (2025a) replicated the effects of aging on response caution (higher value for *b*) and a longer non-decision time *t*0, as with standard DMs in non-conflict tasks before. The new insight gained by applying DMC is that the automatic activation seems to reach its maximum later with increasing age. However, both studies arrive at different conclusions regarding the controlled drift rate $\mu _c$. The source of this difference is not clear, though, and the particular tasks used in these studies or the included age groups and sample size differences might contribute. Future research needs to address these inconsistencies with respect to $\mu _c$ in more detail. Finally, estimating DMC parameters becomes impossible if the conflict task significantly diverges from common conflict tasks, such as the Eriksen flanker and Simon tasks (see Reiber & Ulrich, 2024, for a failure to apply DMC in this research area). If DMC is to be applied in this field, care should be taken to use common conflict tasks.

### DMC and dual-tasking

Conflict effects not only occur in single tasks, but they also occur in multitasking situations, where conflict can be the result of crosstalk arising between the two involved tasks. One example is *backward crosstalk*, first systematically reported by Hommel (1998). Consider an experiment in which a colored letter (e.g., a green or red H or S) is presented as the stimulus. Participants are required to perform two tasks: In Task 1, they are to respond with a manual left or right key press to the color of the letter (Response 1, R1). Subsequently, in Task 2, they are to press a left or right foot pedal in response to the letter’s identity (Response 2, R2). Trials in which R1 and R2 conceptually implicate the same spatial location (e.g., R1: left manual response, R2: left foot pedal press) are referred to as *(R1-R2) congruent (or often: compatible) trials*; when R1 and R2 implicate different spatial features, the trials are referred to as *(R1-R2) incongruent (or often: incompatible) trials*. Backward crosstalk refers to the observation that already Task 1 RTs are shorter in congruent than in incongruent trials, hence the term “backward” crosstalk. This effect has been replicated with many different setups, stimuli, responses (e.g., Ellenbogen & Meiran, 2008, 2011; Hommel & Eglau, 2002; Koob, Dignath, & Janczyk, 2024a; Koob, Durst, Bratzke, Ulrich, & Janczyk, 2020; Koob, Sauerbier, Schröter, Ulrich, & Janczyk, 2024b; Mahesan, Janczyk, & Fischer, 2021; Naefgen & Janczyk, 2019; Renas, Durst, & Janczyk, 2018; Schonard, Ulrich, & Janczyk, 2023; Thomson & Watter, 2013), and age groups (Janczyk, Büschelberger, & Herbort, 2017; Janczyk, Mittelstädt, & Wienrich, 2018a). The multitasking literature considers backward crosstalk most often within extensions of the response selection bottleneck model (Fischer & Janczyk, 2022; Pashler, 1984, 1994; Welford, 1952). According to one account, the Task 2 stimulus automatically activates the corresponding response and this activation interacts with response selection in Task 1 (Janczyk, Renas, & Durst, 2018b). Building on this, Durst and Janczyk (2019) analyzed backward crosstalk with a standard DM and attributed it to different drift rates in congruent and incongruent trials (see also Janczyk et al., 2017). They also wondered whether backward crosstalk is similar to the Eriksen flanker congruency effect, with the Task 2 stimulus being effective similar as flankers are.

This question was recently considered by Koob, Ulrich, and Janczyk (2023a) within the DMC framework. More specifically, these authors conceived the Task 1 response selection as the controlled process within DMC, and the (automatic) Task 2 response activation as the automatic process. In one version, Task 2 response activation followed a pulse-like function, as assumed in the standard DMC. However, it can also be argued that the Task 2 stimulus is never entirely irrelevant (as flankers would be) and it thus might make more sense if the Task 2 response activation increases steadily, perhaps linearly. Both time courses of Task 2 response activation were fitted to data from five published studies (Durst & Janczyk, 2019; Hommel, 1998; Janczyk et al., 2017, 2018a; Koob et al., 2020), and both captured the data qualitatively well. However, a closer inspection of the pulse-like function showed that, in many cases, Task 1 response selection was completed (i.e., the respective diffusion process exceeded the boundary) before Task 2 response activation reached its maximum. Essentially then, this activation followed a monotonically increasing, perhaps asymptotic function (see Fig. 7 in Koob et al., 2023a). Thus, the conclusion was that conflict effects in multitasking situations are not identical to those in standard conflict tasks, like the Eriksen flanker task, rendering DMC less than ideal for modeling interference effects in this context (see also Koob, Ulrich, Ahrens, & Janczyk, 2025b, for a more quantitative approach to this question). Yet, the model provided valuable insights and proved fruitful for formalizing the interplay between Task 1 response selection and Task 2 response activation in dual tasks, thus showcasing a further field of DMC’s application.

Another application of DMC at the intersection of conflict tasks and dual-tasking was presented by Mittelstädt et al. (2023), who investigated how the relevance of distractors influences the strength and timing of cognitive control. To this end, they varied how often distractors in a Simon and Eriksen flanker task required a response. In one condition, the distractor never required a response, while in two others it did so occasionally (in 10% of the trials) or frequently (in 50% of the trials; see also Miller & Durst, 2014, for a similar experimental approach). At the behavioral level, greater distractor relevance led to increased mean RTs to the target and overall larger congruency effects. Additional DMC analyses revealed that both the boundary parameter, *b*, and the non-decision time, *t*0, increased with distractor relevance. More interestingly, in terms of DMC, both the amplitude, *A*, and $\tau$ of the automatic process increased, while $\mu _c$ decreased. This pattern indicates a clear trade-off between controlled and automatic processing, highlighting strategic adjustments in cognitive control in the context of conflict tasks and dual-tasking.

### Summary

Since its introduction, DMC has been widely applied to various conflict tasks, primarily, however, to the Simon and Eriksen flanker tasks within cognitive psychological research. Apart from providing insights into standard congruency effects, DMC has also helped gain insights into the underlying mechanisms of the congruency sequence effect (Koob et al., 2023b; Luo & Proctor, 2022) and the proportion congruency effect (Kelber et al., 2025b; Luo et al., 2023). Here, DMC points to a prominent and important role of the strength of distractor processing/suppression (see also Gheza & Kool, 2025, for a similar conclusion from a different modeling background). No study exists, to the best of our knowledge, that has applied DMC to item-specific proportion congruency effects (Jacoby, Lindsay, & Hessels, 2003). Future modeling attempts with DMC should include it as well, as it provides a core phenomenon, together with the proportion congruency effect and the congruency sequence effect, that any cognitive control theory and model should account for. DMC has further been used to address modeling neutral trials and the distinction between facilitation and interference (Evans & Servant, 2022; Smith & Ulrich, 2024, 2025), and conflict effects in dual-tasking (Koob et al., 2023a, 2025b; Mittelstädt et al., 2023).

Beyond cognitive psychology, DMC has been used in aging research (Evans & Servant, 2022; Kelber et al., 2025a) and in individual differences research. There, the focus of DMC applications is on model-based analyses of the congruency effect’s reliability (Hedge et al., 2022; Hedge, Powell, & Sumner, 2018b). Assessments of test-retest reliability of parameter estimates (Hedge et al., 2019) and their associations with other personality variables (Hedge et al., 2020) exist, but are rare to date. DMs have also been used in clinical and neuropsychological research (e.g., Metin et al., 2013; White, Ratcliff, Vasey, & McKoon, 2010a; Zhang et al., 2016; see White, Ratcliff, Vasey, & McKoon, 2010b, for a broader discussion of DMs’ benefits in this context). Yet the explicit use of DMC is rather rare. One study by Servant et al. (2018; see also Mann et al., 2023) analyzed data from a Simon task in patients with Parkinson’s Disease. Another recent study by Patel, Green, Barch, and Wynn (2025) reported data from a Stroop-like task in groups of schizophrenia and bipolar disorder patients. A final mention is that DMC has also been used to investigate the impact of sleep deprivation (Luo et al., 2024) and mild acute stress (Shields, Rivers, Ramey, Trainor, & Yonelinas, 2019) on performance in conflict tasks.

## Technical aspects and recommendations

The previous sections introduced DMC and reviewed fields of its application. We now turn to the more practical and technical details concerning the application of DMC. Hence, in this section, we provide an overview of fitting procedures and software solutions, parameter recovery, and questions we are often asked for guidance on. These include whether *a* should be estimated or fixed at $a=2$, and the importance of starting point variability. Hence, this section is particularly important for those already using DMC, who want to gain more insights into advances and practical recommendations.

### Fitting procedures, software, and respective advantages and disadvantages

Fitting DMC to (observed) data means to find the set of parameters with which DMC best predicts the observed data pattern. As with any model, this requires (at least) three considerations: the method to derive DMC’s predictions, an objective function that quantifies the discrepancy (or similarity) between the observed data and the model predictions, and finally, an optimization routine that explores the parameter space for those parameter sets that yield the best match.^11^ In theory, these three aspects can be combined arbitrarily; however, certain combinations go along with each other more naturally in practice. We begin with reviewing the typical approaches to fit DMC including some advantages and disadvantages of the respective approaches. This is followed by a description of the existing software packages to fit DMC. Readers familiar with fitting cognitive models might want to skip to the section “Software implementations”.

#### Monte Carlo simulation and summary statistics

The original approach to fit DMC was to derive DMC’s predictions via Monte Carlo simulations and to compare observed and predicted data using summary statistics of observed and simulated RTs (Ulrich et al., 2015). Specifically, to gain model predictions for a given set of parameters, a large number of evidence accumulation processes are simulated. By recording the time when each process reached the boundary (and the response choices), and adding the non-decision time, this yields an approximation of the RT distribution. To simulate a single process, researchers often use the flexible Euler–Maruyama method, which approximates Equation 6 by a difference equation:

10 $$\begin{aligned} \textbf{X}(t + \Delta t)&\!=\! \textbf{X}(t) + (\mu _c \!+\! \mu _a(t)) \Delta t + \sqrt{\Delta t}\cdot \sigma \textbf{Z}(t) \, \,. \end{aligned}$$

Here, $\textbf{Z}(t)$ is the standard normal distribution, $\mu _c$ the drift rate of the controlled process, $\mu _a(t)$ the drift rate of the automatic process (resulting from *a*, $\tau$, and *A*; see Fig. 7), and $\Delta t$ the time step size, that is, the increment in the domain of time. The main difference between Eqs. 10 and 6 is that the former focuses on the momentary change of the decision process over a small time step $\Delta t$, and allows to flexibly realize the decision process over time.^12^

In the context of DMC, a step size of $\Delta t = 1\text {ms}$ and about 10,000 to 100,000 simulated trials per congruency condition have been used (e.g., Koob et al., 2023a; Luo & Proctor, 2022; Mackenzie & Dudschig, 2021; Servant et al., 2016; Ulrich et al., 2015; White, Servant, & Logan, 2018).

Given that the Monte Carlo approach gives predictions in a similar format as the observed data, both predicted and observed data are compared using the same descriptive statistics; in practice, often quantiles, CAFs, and delta functions. Several summary statistics exist to quantify the difference between observed and predicted descriptive statistics, and have been used with DMC. The *Root Mean Squared Error* (*RMSE*; e.g., Ellinghaus et al., 2024b; Held et al., 2024; Kelber et al., 2025a; Koob et al., 2023a; Mittelstädt et al., 2023; Mittelstädt et al., 2022; Ulrich et al., 2015), the $G^2$ statistic (e.g., Hübner & Töbel, 2019; Luo & Proctor, 2022; Luo et al., 2023; Mahani et al., 2019; Servant et al., 2016), the $\chi ^2$ statistics (e.g., White et al., 2018), $\Lambda$ (Hedge et al., 2019), and *SPE* (Hübner & Pelzer, 2020). Note that not all of them are specific to DMC, but are quite common in statistics and modeling. We find the best-fitting parameters by minimizing the respective statistic, and the typical algorithms in this regard are discussed further below. More detailed information about the summary statistics is provided in the Electronic Supplement D.

#### Fitting individual or aggregated data

With summary statistics, DMC can be fitted to each individual’s data (e.g., Hedge et al., 2019; Servant et al., 2016) or to data averaged across individuals (e.g., Hübner & Töbel, 2019; Kelber et al., 2025a; Mahani et al., 2019; see also Ulrich et al., 2015, where data were averaged across subgroups of all participants). In the former case, parameter estimates are obtained for each individual and can then be subjected to further analyses (e.g., comparisons across groups or conditions). In the latter case, one first averages the individual summary statistics of quantiles and CAFs across participants, and then fits DMC to the aggregated data once, yielding a single set of parameters.

Aggregating the data has the advantage of highly reducing computational burden, as DMC only needs to be fitted once. Additionally, individual data can be very noisy, especially when trial numbers are moderate, resulting in unreliable parameter estimates (Cohen, Sanborn, & Shiffrin, 2008). In such cases, averaging can help to obtain a clearer picture of the shape of the (average) RT distribution and thus lead to more stable parameter estimates. However, aggregating data requires some caution. Parameter estimates obtained from aggregated data do not necessarily match the average parameter estimates obtained with individual’s data (Estes & Todd Maddox, 2005; Rouder & Speckman, 2004). Moreover, averaging implicitly assumes that the same model generated all data and that it operates identically for each individual, and that the parameters estimated from the aggregated data reflect the parameter values of each individual. A bias resulting from this aggregation may often be tolerable, and this may be assessed by employing both approaches.

The choice between fitting individual or aggregated data may depend on the goal. When the goal is to test hypotheses about individual differences or to perform inferential statistics across participants, fitting DMC to individual data is essential. However, when the goal is primarily descriptive or exploratory, such as demonstrating the qualitative match between model predictions and data or comparing models, fitting aggregated data can be pragmatic and informative. Regardless of the choice, researchers should be aware of the assumptions and potential limitations associated with each method. Overall, though, fitting DMC to each individual appears as the more common approach (as was done in, e.g., Ellinghaus et al., 2024b; Evans & Servant, 2020, 2022; Hedge et al., 2019, 2022; Kelber et al., 2023; Koob et al., 2023b; Mittelstädt et al., 2023; Servant et al., 2016, 2018). An alternative to fitting individual data separately is hierarchical fitting within the Bayesian framework, as will be introduced below in more detail.

#### Using full-range maximum likelihood instead of summary statistics

Instead of using DMC’s predicted RT data by converting them into quantiles and CAFs, one can also directly use the (full) predicted distribution of RTs for *maximum likelihood estimation (MLE)* of the parameters. MLE is one of the most widely used and principled concepts in statistics, for example, since MLE reaches the lower bound of the Cramér-Rao-inequality. The goal of MLE is to find those parameters that maximize the likelihood function (or, from a practical perspective, which minimize the negative log-likelihood function). More details are provided in the Electronic Supplement E for interested readers.

While MLE is a common method for fitting standard DMs (Voss et al., 2015), it has rarely been used for DMC. As far as we are aware, only Janczyk et al. (2025) and Koob et al. (2023b) have applied it so far. One reason is that, until recently, DMC predictions were predominantly obtained with Monte Carlo simulations as described above. Consequently, the predicted data obtained from the simulation were RT data and not directly the PDFs. Of course, one can approximate the PDFs from the simulated RTs via kernel density estimation (Evans & Servant, 2020, 2022; Turner & Sederberg, 2014), but approaches based on summary statistics dominate the literature. However, more sophisticated methods are now available and allow to numerically compute these PDFs (Richter, Ulrich, & Janczyk, 2023; Shinn, Lam, & Murray, 2020, making MLE straightforward for DMC (see below for more information).

#### Typical algorithms for classical optimization

Above, we have summarized classical approaches for quantifying how well observed data from conflict tasks and DMC predictions match. A core ingredient that helps us find the best-fitting model parameters is an algorithm that minimizes the respective *objective function*. In most DMC applications, researchers have used either Nelder–Mead (e.g., Hedge et al., 2019, 2020; Hübner & Pelzer, 2020; Mahani et al., 2019; Servant et al., 2016, 2018; White et al., 2018) or Differential Evolution (e.g., Held et al., 2024; Janczyk et al., 2025; Kelber et al., 2025a; Koob et al., 2023b; Mittelstädt et al., 2022, 2023).

Nelder–Mead (Nelder & Mead, 1965) is a simple yet widely used optimization method. It works by moving and reshaping a simplex (a geometric figure) across the parameter space to improve the fit to the data. Nelder–Mead is also considered a “downhill” algorithm. To illustrate, suppose we want to estimate two parameters of DMC. Each parameter combination yields a value of the objective function, which we can visualize as a surface over a multi-dimensional parameter space. This surface curves down toward a minimum where the optimal parameters lie. Nelder–Mead begins by using the provided starting values to place a simplex on this surface, which, in two dimensions, is a triangle. From there, the minimization routine can be imagined as the triangle tumbling down the surface; once it comes to rest in the valley, the optimal parameter values have been determined (see also Farrell & Lewandowsky, 2018, for an accessible introduction to Nelder–Mead). Importantly, however, Nelder–Mead is prone to local minima. Depending on the starting values, it can easily descend into an area of small objective function values that are low, albeit not the lowest overall. In other words, the algorithm might get stuck in a local, but not in the global minimum. This might indeed happen with DMC, which is a relatively complex DM with many parameters that often affect model predictions similarly (see again Fig. 5). To counteract this problem, researchers almost always run Nelder–Mead multiple times with different starting values or precede the estimation procedure with a systematic grid search (Hübner & Pelzer, 2020; White et al., 2018).

Differential Evolution is a population-based algorithm (Storn & Price, 1997). It begins with a large set of random parameter combinations scattered across the objective function, the so-called “population”. At each iteration, it randomly selects two parameter combinations (i.e., two “members” of the population) and adds the (scaled) difference between them to the parameter values of a third member. If the resulting parameter combination yields a better model fit, it replaces the old one.^13^ Because Differential Evolution involves an entire population that explores the objective function, it is very robust, even with many local minima. Its main drawback is that it can be computationally expensive, as the objective function needs to be evaluated for each member of the population in every iteration.

#### (Hierarchical) Bayesian estimation

A final approach we discuss here does not rely on classical minimization of an objective function, but instead uses the Bayesian framework to guide parameter inference. During Bayesian estimation, model parameters are treated as random variables, and inference proceeds by updating prior beliefs about these parameters in light of observed data (see Farrell & Lewandowsky, 2018; Kruschke, 2014; Lee & Wagenmakers, 2014, for introductory texts). Specifically, Bayesian inference also depends on the probability distribution of the data given a set of parameters, $f(y \mid \theta )$, just like MLE does. However, it goes further by multiplying $f(y \mid \theta )$ with a prior probability distribution, $f(\theta )$, which expresses beliefs about the parameters before observing the data. The product yields the (unnormalized)^14^ posterior distribution

11 $$\begin{aligned} f(\theta \mid y) \propto f(y \mid \theta ) \cdot f(\theta )\,. \end{aligned}$$

This posterior distribution provides us with information about the probability of the parameters given the data. This is an important difference to classical MLE, which provides us with information about the likelihood of the data given the parameters. It is worth mentioning that the prior knowledge is not only a means-to-an-end to derive the posterior distribution. It helps to constrain estimates and guide inference, especially when data are sparse or noisy.

Another key difference to MLE is that Bayesian inference does not yield a single, estimated parameter set. Instead, it aims to characterize the full posterior distribution, $f(\theta \mid y)$. As a result, Bayesian inference inherently provides information about the most probable parameter values $\theta$, and uncertainty estimates for them.

One of the major advantages of the Bayesian approach is that it naturally extends to hierarchical estimation, where parameters are estimated at multiple levels: at the individual level (i.e., for each participant) and at the group level. This approach has become increasingly popular in recent years, as it constrains individual-level parameters to follow group-level distributions (Shiffrin, Lee, Kim, & Wagenmakers, 2008). Formally, the hierarchical structure can be expressed as

$$f(\Theta , \Phi \mid D) \propto f(D \mid \Theta ) \cdot f(\Theta \mid \Phi ) \cdot f(\Phi )\,.$$

Here, $\Theta$ represents all model parameters across individuals, $\Phi$ contains the group-level parameters, and *D* represents the observed data across all participants. In a hierarchical setting, the goal is to estimate the posterior distribution of both individual- and group-level parameters given all available data. This involves the probability distribution of the data given individual-level parameters, $f(D \mid \Theta )$, the distribution of individual parameters given the group-level parameters, $f(\Theta \mid \Phi )$, and the prior over the group-level parameters, $f(\Phi )$.

Because the hierarchical approach considers all participants at the same time, “shrinkage” of individual parameter estimates toward the group-level mean occurs. This helps stabilize individual estimates and reduces the risk of overfitting. The other, more classical approaches discussed above, can only estimate each participant’s parameters independently. This can lead to (a) larger variability in individual estimates, and (b) individual parameter sets to “wander off” relative to the group mean. In our applications of DMC, we have often observed this, particularly for the time point of the maximum activation within the automatic channel, that is, $\tau$ if $a=2$, and the boundary *b*.

While the Bayesian approach has several advantages, it is not easy to implement: In addition to the probability distribution $f(y \mid \theta )$, one needs to specify a reasonable prior distribution and use a Markov Chain Monte Carlo (MCMC) sampler to explore the posterior distribution (Turner, Sederberg, Brown, & Steyvers, 2013; Van Ravenzwaaij, Cassey, & Brown, 2018). Moreover, exploring the posterior requires many iterations, making Bayesian inference generally more computationally intensive than the other approaches described above. As with MLE, obtaining accurate PDFs to calculate the likelihood was, until recently, a technical barrier. With new software tools, this limitation is diminishing, and Bayesian (hierarchical) modeling of DMC is likely to become more feasible and widespread in the future. To date, Bayesian inference has rarely been used with DMC, with the notable exceptions provided by Evans and Servant (2020, 2022).

#### Software implementations

We now provide an overview on existing software to fit DMC to empirical data. While many (early) applications of DMC relied on custom code (e.g., Evans & Servant, 2022; Hedge et al., 2019; Hübner & Töbel, 2019; Koob et al., 2023a; Servant & Evans, 2020; Servant et al., 2016), several dedicated software solutions have emerged in recent years that allow researchers to apply DMC more easily. Here, we focus on software that provides DMC out-of-the-box, but many excellent packages can be used alternatively, such as |pyDDM| (Shinn et al., 2020), |CHaRTr| (Chandrasekaran & Hawkins, 2019), |flexDDM| (LaFollette, Fan, Puccio, & Demaree, 2024), or |BayesFlow| (Radev et al., 2023).

One of the earliest and most widely used packages is |DMCfun|, available for both R and Python (Mackenzie & Dudschig, 2021). It implements the original approach of deriving model predictions via Monte Carlo simulation and parameter optimization via summary statistics. Supported objective functions include *RMSE*, $\chi ^2$, $G^2$, and *SPE*, and optimization can be done via (bounded) Nelder–Mead or Differential Evolution. A drawback of the simulation approach is the introduction of random variability into the predictions and the objective function values, which can hinder optimization. More importantly, however, simulation is considerably less efficient than more sophisticated numerical methods utilized in the packages listed below (Richter et al., 2023). Model customization is possible, albeit within the limits of pre-built options provided by the package. Nevertheless, |DMCfun| remains a very user-friendly, well-documented package, and it is often the first option researchers try.

A more recent Python package is |pyBeam| (Murrow & Holmes, 2024b), which derives model predictions by numerical methods based on the Kolmogorov Forward Equation (see also Richter et al., 2023; Shinn et al., 2020). Parameter estimation is implemented via a (non-hierarchical) Bayesian approach using the Python package |PyMC| (Salvatier, Wiecki, & Fonnesbeck, 2016) and an MCMC sampler related to the logic of Differential Evolution (ter Braak, 2006; ter Braak & Vrugt, 2008). Advantages of |pyBeam| include support for model customization and, in particular, very high computational speed. A limitation is that it does not currently include functionality to compute standard summary statistics for DMC, like CAFs or delta functions. Building on |pyBeam|, the R package |ream| (Hartmann & Murrow, 2024) provides access to the same mathematical approach for R users. Thus, it is also optimized for speed and supports model customization. However, it currently only provides functions to compute the PDFs/CDFs of DMC, the log-likelihood, or to sample trials from it, but users need to link |ream|’s output to a minimization algorithm and code their own functions to derive CAFs and delta functions.

The R package |dRiftDM| (Koob et al., 2025a) lies in between |DMCfun| and |pyBeam|/|ream|. Like the latter two, it derives model predictions via the Kolmogorov Forward Equation (see Richter et al., 2023), but it also includes out-of-the-box functionality for fitting DMC to multiple individuals and for extracting typical DMC summary statistics. It supports model customization and parameters are estimated via MLE or the minimization of the *RMSE* statistic. Starting with Version 0.3.0, it also provides access to both hierarchical and non-hierarchical Bayesian estimation using an MCMC sampler based on Differential Evolution (ter Braak, 2006; Turner et al., 2013). On the downside, |dRiftDM| is currently not as optimized for computational speed as |pyBeam| or |ream|, although it is sufficiently fast for most applications.

At present, no comprehensive study exists that systematically compares all these packages and estimation methods for DMC. Still, all packages and approaches will do a reasonable job. The specific software choice and modeling approach will thus heavily depend on the individual researchers and their skills. If users are familiar with R and less programming experienced, |dRiftDM| and |DMCfun| will have the most appeal. If users want to maximize speed, then |ream| or |pyBeam| will be more interesting. If Bayesian inference is desired, researchers will lean toward |pyBeam| or |dRiftDM|.

There are, however, some general recommendations: Approaches that rely on the complete PDF are more efficient for outlier-free data than approaches based on summary statistics. Methods that constrain parameters, either by enforcing boundaries on the search space or by specifying priors, can increase estimation precision. Algorithms based on Differential Evolution are less susceptible to local minima and thus lead to more robust parameter estimates. Finally, hierarchical methods (which will surely be more common in the future), can help constrain parameter estimation by considering all participants simultaneously. These recommendations hold for DMC as much as for any other DM (Lerche, Voss, & Nagler, 2017; Ratcliff & Tuerlinckx, 2002; Wiecki, Sofer, & Frank, 2013). Apart from the specific choice of the fitting approach, however, researchers will also have to make sure that they collect enough trials to ensure a reliable estimation of DMC’s parameters.

### Trial numbers and parameter recovery

An important consideration when designing experiments to which DMC (or any other model) will be subsequently applied concerns the number of trials per congruency condition and participant. Studies that examine the distributional properties of RTs irrespective of any model fitting, for example, by examining delta functions, require more trials than those just comparing cell means. Indeed, in the original study with delta functions (De Jong et al., 1994), the first two experiments involved three sessions with 16 blocks of 48 trials per session, resulting in over 1000 trials per congruency condition and participant. While pragmatic considerations (e.g., working with special populations such as children or clinical groups) will contribute to such decisions, parameter recovery studies (see below) suggest a general principle: the more trials, the better.

That said, an important question is, What is the recommended (or minimum) number of trials needed for subsequent reliable parameter estimation? Considering published empirical data, a wide range of trial numbers is evident. As noted by Lerche et al. (2017) with reference to the standard DM, there “has been a huge variation in the numbers of trials used for previous diffusion model experiments, ranging from less than 100 to several thousands of trials per participant” (p. 514). For example, the original DMC study from Ulrich et al. (2015) used 168 trials per congruency condition and participant. In contrast, Ambrosi et al. (2019) investigating conflict processing within a kindergarten aged sample used just 48 trials per congruency condition and participant, whereas Servant et al. (2016) had 1152 trials per congruency condition and participant.^15^ White et al. (2018) suggest that, although there is considerable variability, a range of 50–500 trials per congruency condition reflects typical real-word experimental practices.

Several studies have systematically investigated parameter recovery properties of DMC via large-scale simulations across a wide range of trial numbers (Evans & Servant, 2022; Hedge et al., 2022; Hübner & Pelzer, 2020; Janczyk et al., 2025; Luo et al., 2023; Murrow & Holmes, 2024b; White et al., 2018). Despite some methodological variations, the general process unfolds in three steps. First, a number of simulated data sets with known parameters is generated. These data sets consist of *N* trials each, with parameter values sampled from defined distributions spanning the typical parameter space of the model under investigation. These simulated datasets serve as stand-ins for empirical observations, or the “observed” data. Second, the model is fitted to these synthetic data. Third, it is evaluated how precisely the estimated parameters match those parameter values that were originally used to generate the data. This is typically quantified using a correlation between the generating and the estimated parameter values. High correlations indicate good parameter recovery, demonstrating that parameter estimates are reliable. For a computational model, this is one of the most important prerequisites to trust parameters estimated from empirical data.^16^

The first comprehensive parameter recovery of conflict DMs, including DMC, was done by White et al. (2018); the other models were those by White et al. (2011), and Hübner et al. (2010). The authors investigated parameter recovery with $N\in \{50, 100, 200, 500, 1000, 5000\}$ trials per congruency condition. Generating parameters were sampled from uniform distributions representing realistic ranges for each model. Each model was fitted 20 times using Nelder–Mead and different starting values also drawn from a random distribution. Overall, parameter recovery decreases with increasing number of free model parameters, reflecting greater estimation difficulty in more complex models. Regarding specific parameters for DMC, differences across the parameters became observable. Using the authors’ correlation-based classification of “poor” ($r < .5$), “fair” ($.5< r < .75$ ), “good” ($.75< r < .90$), and “excellent” ($r > .9$), the recovery of $\mu _c$, *b*, and *t*0 was good to excellent, even with relatively small trial numbers ($N\le 200$). However, recovery of conflict-related parameters (*A*, $\tau$, and *a*) was not always ideal. Regarding *A*, recovery was “fair” for small trial numbers ($N\le 200$) and reached “good” for $N\ge 500$. Regarding *a* and $\tau$, recovery remained rather poor to fair, even for unrealistically large trial numbers (e.g., $N \ge 1000$). Importantly, however, one has to consider that *a* and $\tau$ jointly determine the time point of the maximum activation within the automatic route, that is, $t_{\text {max}} = (a - 1) \tau$. White et al. (2018) therefore also reported recovery properties for $t_{\text {max}}$, which was recovered well even for relatively small trial numbers (e.g., $r = .91$ with $N = 200$). Consequently, when both *a* and $\tau$ are estimated, each can only be recovered poorly, whereas their joint influence on $t_{\text {max}}$ can be recovered well. This makes sense because of a certain trade-off between $\tau$ and *a*. Particularly, several combinations of $\tau$ and *a* can lead to very similar predictions (i.e., small $\tau$ and large *a* values lead to predictions similar to those from large $\tau$ and small *a* values).

Two other parameter recovery studies (Hübner & Pelzer, 2020; Murrow & Holmes, 2024a) support the conclusions of White et al. (2018). For example, Hübner and Pelzer additionally introduced an initial grid search to circumvent potential issues related to local minima when using Nelder–Mead. Recovery improved with the grid search technique, but the overall pattern remained the same: While the parameters $\mu _c$, *t*0, and *b* were recovered very well, recovery of the conflict-related parameters *A*, *a*, and $\tau$ was again worse. While recovery for *A* was “good” for $N \ge 200$, *a* and $\tau$ required at least $N = 500$ to $N = 5000$ trials per congruency condition to be recovered “good.” Yet again, recovery for $t_\text {max}$ was already “good” with $N=100$.

Further studies did not estimate *a* and $\tau$ simultaneously, but fixed $a = 2$ during the simulation procedure (Evans & Servant, 2022; Hedge et al., 2022; Janczyk et al., 2025). Hedge et al. (2022, Appendix B) report a parameter recovery study for DMC with trial numbers consistent with their empirical data sets (around $N=300-600$ per congruency condition). Parameter recovery was “fair” to “good” for $\mu _c$, *b*, *A*, *t*0, and $s_{t0}$, and, to a lesser extent, $\alpha$. However, results for $\tau$ were highly heterogeneous. In some cases, correlation-based recovery for $\tau$ was “good” (e.g., $r = .86$), whereas in others it was even slightly negative (e.g., $r = -.08$).

Evans and Servant (2022) also report a parameter recovery study for their extension of DMC where the amplitude *A* could differ between congruent and incongruent trials. To assess parameter recovery, they employed a Latin hypercube sampling design to generate a number (3000 in the first, 10,000 in subsequent studies) of simulated parameter sets. Critically, from these simulated parameter sets, a subset was selected based on a criterion involving several RT and accuracy properties (e.g., 400 ms < mean RT < 2500 ms; 0.6 < Accuracy < 1). Parameter estimation was done in the Bayesian framework. In general, recovery performance in this study was very good across the full range of trial numbers investigated ($N\in \{200, 500, 1000\}$ per congruency condition). Although the recovery of $\tau$ was slightly worse than for the remaining parameters, it was still “good” (with correlation values of approximately .85). The authors also note that the parameter recovery error for $\tau$ increased as the generating value increased, suggesting that moderate and high values of $\tau$ are somewhat indistinguishable (matching with simulations in the dual-task context, see Koob et al., 2025b). One might suspect that, with sufficiently large values for $\tau$, the boundary may already have been exceeded and thus there is not much of a difference between $\tau$ values beyond this point.

Finally, Janczyk et al. (2025) also report a parameter recovery for DMC (as well as RDMC) using |dRiftDM|. Data were generated from relatively broad uniform distributions and fitting was done with Differential Evolution and MLE. Trial numbers covered a range of $N\in \{\text {200, 500, 1000, 10,000}\}$ per congruency condition. Parameter recovery was “good”, even at low trial numbers ($N = 200$), for $\mu _c$, *A*, *b*, and *t*0. While parameter recovery for $\tau$ was “fair” with $N = 200$, it was good with $N \ge 500$.

The general pattern of all these different parameter recovery studies seems that DMC’s properties in this regard are good to excellent for the majority of parameters even with relatively small trial numbers. The rather consistent exceptions are $\tau$ and *a*. When both parameters are estimated, recovery for each parameter is less than ideal (Hübner & Pelzer, 2020; Murrow & Holmes, 2024a; White et al., 2018), while the time point of the maximum activation within the automatic route, $t_\text {max}$, is still recovered well (Hübner & Pelzer, 2020; White et al., 2018). When fixing $a=2$, thus setting $t_\text {max} = \tau$, recovery for $\tau$ is generally improved (Evans & Servant, 2022; Janczyk et al., 2025), particularly when focusing on data sets where mean RTs and accuracy were in ranges found in empirical data (see Evans & Servant, 2022; though see also Hedge et al., 2022, where recovery of $\tau$ sometimes failed remarkably).

Based on the aforementioned studies, we currently judge $N = 100-200$ trials per congruency condition as the absolute minimum recommendation. With such trial numbers, recovery for *b*, $\mu _c$, and *t*0 is at least “good” (including $t_\text {max}$ if *a* and $\tau$ are both estimated). Additionally, *A* is recovered “good” with about $N = 200$ trials. If the focus is also on $\tau$ (with $a=2$), researchers should aim for $N = 200-500$ trials per congruency condition. Finally, if researchers really want both *a* and $\tau$ to be estimated precisely, then very robust estimation procedures are required with very high trial numbers (of at least $N = 500$ per congruency condition and above; Hübner & Pelzer, 2020). In addition, according to our experience with DMC, including $s_{t0}$ slightly worsens the recovery of other parameters, but still yields a better qualitative fit of DMC to data. This would also be an argument for using even larger trial numbers ($N>500$). Naturally, the more trials, the better.

While the previous literature review on the recovery performance of DMC provides some guidance on trial numbers, several issues remain open. A systematic investigation is lacking on how the decision to fix or estimate *a*, as well as the exclusion of data with unrealistic RTs and accuracy values, affects recovery performance. The same applies to systematic investigations of different ranges of $\tau$ and their influence on parameter recovery. Additionally, recovery performance in cognitive models is often worse when accuracy is high (see, e.g., Lüken, Heathcote, Haaf, & Matzke, 2025, for a recent simulation study using the standard DM), that is, when error data are missing that usually help to constrain the model during estimation. Regarding DMC, it is currently unclear how, and to what extent, this issue of high accuracy affects recovery.

### Estimating *a* or fixing $a=2$?

An important, yet somewhat unresolved, issue is whether the shape parameter *a* shall be fixed to $a=2$ or be estimated freely. In the original article by Ulrich et al. (2015), DMC was introduced with $a=2$, providing a more parsimonious description of the pulse-like automatic process. However, in subsequent applications of DMC, *a* was sometimes estimated freely (e.g., Ellinghaus et al., 2024a; Mittelstädt et al., 2023; Servant et al., 2016), and sometimes not (e.g., Evans & Servant, 2022; Kelber et al., 2023; Koob et al., 2023b; Servant & Evans, 2020).

The arguments for estimating *a* freely are two-fold. First, the expected time-course of the automatic activation becomes more flexible and can thus offer more nuanced descriptions and insights into task-irrelevant processing (beyond $t_{\text {max}}$, see Fig. 4 and also Servant et al., 2018). Additionally, *a* and $\tau$ jointly determine $t_\text {max} = (a-1) \tau$, and the parameter recovery studies summarized above demonstrate that estimating both *a* and $\tau$ freely results in good recovery properties for $t_\text {max}$, even for small trial numbers (Hübner & Pelzer, 2020; White et al., 2018). Arguably, $t_\text {max}$ is often more interesting to psychologists than *a* or $\tau$ alone. The arguments for fixing $a=2$ emphasize model parsimony and that estimating both *a* and $\tau$ reliably requires elaborated estimation procedures and (unrealistically) large trial numbers (Hübner & Pelzer, 2020). Consequently, when estimating both *a* and $\tau$ freely, one often trades a more reliable estimation of $t_\text {max}$ for non-reliable estimates of *a* and $\tau$. Additionally, when both *a* and $\tau$ are estimated, there are more parameter trade-offs. In fact, already White et al. (2018) noted that fixing *a* increases the recovery for the amplitude *A*.

An additional, rather technical argument for fixing $a=2$, relates to the (time-dependent) drift rate of the automatic process, $\mu _a(t)$, that is, the first derivative of $\mathbb {E}[\boldsymbol{X_a}(t)]$ with respect to time *t* (the resulting Eq. 5 is reprinted here for convenience):

12 $$\begin{aligned} \mu _a(t) = A e^{-\frac{t}{\tau }} \cdot \left[ \frac{t e}{(a-1) \tau } \right] ^{a-1} \cdot \left[ \frac{a-1}{t} - \frac{1}{\tau } \right] \,. \end{aligned}$$

From this equation, it becomes clear that the derivative does not exist at $t = 0$, as it implies division by 0 (in the second parentheses). However, for some cases, the derivative exists in the limit for $t \rightarrow 0$, which we can use for a continuous extension. By reformulating the equation, one can eliminate *t* in the denominator, and reveal the boundary condition under which the limit does not exist (see also the Electronic Supplement F for a step-by-step derivation of this equation):

13 $$\begin{aligned} \mu _a(t) = A e^{-\frac{t}{\tau }} \cdot \left[ \frac{t e}{(a-1) \tau } \right] ^{a-2} \cdot \left[ \frac{\tau e (a-1) - t e}{(a-1) \tau ^2} \right] \,. \end{aligned}$$

The crucial part is the exponent $(a-2)$ for the first parenthesis. Specifically, for any value $a < 2$, the exponent becomes negative. Because the parentheses become 0 if $t \rightarrow 0$, one would have to take 0 to the power of something negative, which is undefined (e.g., for $a = 1.5$ and $t = 0$, the first parenthesis of $0^{1.5-2} = 0^{-0.5}$ is undefined).

**Fig. 8:**
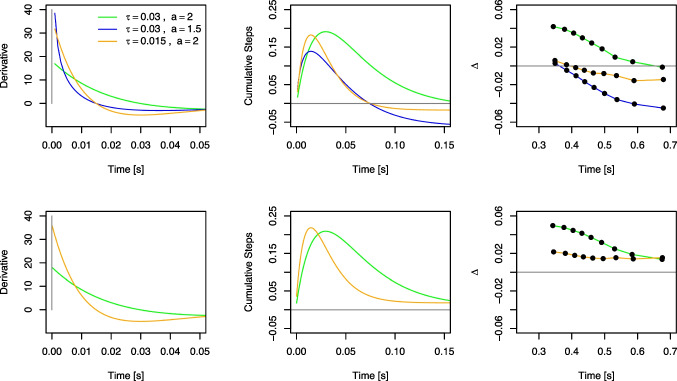
An illustration of potential problems when approximating the time-course of automatic processing. The *left-most panels* show the derivative of the Gamma distribution function (i.e., of the expected activation within the automatic route). The *vertical gray line* marks $t= 0$. The *middle panels* show the (scaled) cumulative sum of the respective derivatives, approximating the expected time-course of the Gamma distribution function. The *gray horizontal line* at 0 highlights the undershoot. The *right-most panels* show simulated delta functions. These were simulated using the Euler–Maruyama method. The upper and lower rows of the plot show the cases of skipping and not skipping the first value of the first derivative, respectively. Note that $a=2$ was fixed for the lower row. The other parameters were $\mu _c = 3$, $A = 0.2$, $b = 0.6$, $\sigma = 1$, and $\Delta t = 0.001$

Why is this important? Oftentimes, DMC’s predictions require the derivative of the rescaled Gamma distribution function (see, e.g., Eq. 10). Crucially, the derivative of this function is very large in the beginning (see Figs. 7 and 8), such that there is a large momentary rate of change in evidence accumulation within the automatic route. This momentary rate of change is larger the smaller *a* or $\tau$, reflecting the fact that the automatic process is pulse-like, rising quickly toward one of the two boundaries in the first few steps. There are now two important aspects to consider.

First, given the large initial momentary change, the step size of the first few steps in the time domain, $\Delta t$, is very important. If the step size is large, and the momentary change is huge, then we will not accurately describe the dynamics of automatic processing and DMC’s predictions will be particularly inaccurate.

Second, because the derivative at $t= 0$ does not exist, we will have to ignore the momentary change in this very first step of evidence accumulation, which increases numerical inaccuracy even further (see also Fig. 9 in Luo et al., 2023). This is visualized in the upper half of Fig. 8. The left panel shows the derivative of the Gamma distribution function for relatively small *a* and $\tau$ values. The middle panel shows the approximated time-course of automatic processing in congruent trials, derived by accumulating the derivative across time when skipping the first step at $t=0$.^17^ The important result is that earlier maxima of the function (the orange and blue lines), that is, of the activation within the automatic route, lead to a slight undershoot of the automatic process. Consequently, the predicted delta functions yield values that reach into the negative area. Additionally, this undershoot is particularly extreme for small *a* values. When the derivative is continuously extended to $t=0$ for $a = 2$, however, the numerical error is slightly reduced (see the lower half of Fig. 8), although the problem is never abolished entirely. For example, the orange curve in the lower middle panel never fully reaches 0.

In sum, the derivative of the Gamma distribution function is large in the beginning and does not exist at $t=0$. This increases numerical inaccuracies in the very first steps and can alter DMC’s predictions considerably. When fixing $a = 2$, we gain two advantages. First, the derivative of the gamma distribution function is less extreme in the beginning. Compare, for example, the orange and blue curve in the upper row of Fig. 8, both of which model the same $t_{\text {max}}$. Second, we can use the continuous extension of the derivative at the very first step to further increase numerical accuracy (see the lower row).

Should one fix $a = 2$ or not now? There is no general answer in our opinion. Estimating both *a* and $\tau$ is difficult, but bears the advantage of a reliable estimate of $t_\text {max}$. Yet again, estimating both *a* and $\tau$ is less parsimonious and can lead to wrong DMC predictions due to numerical inaccuracies that appear during the first few steps of the automatic process. Critically, however, this numerical error will not always be the same. If *a* and $\tau$ are rather large, the momentary rate of change of the automatic process is relatively small in the beginning, and the numerical error will not be severe. This would, for example, be the case in the Eriksen flanker or Stroop task, but not in the Simon task. Additionally, the numerical error will depend on the step size $\Delta t$ used for deriving DMC’s predictions. With a relatively small step size in the beginning, numerical accuracy is better ensured. If, however, relatively large step sizes were chosen in the beginning, then numerical inaccuracies will likely result. Software solutions differ in this regard. For example, |DMCfun| always uses the same step size. |pyBeam|, |ream|, and |dRiftDM| use a dynamic step size, with smaller step sizes in the beginning than in the end. Therefore, it will depend on the conflict task at hand and the specific software implementation used when deciding about fixing *a* or not. In any case, we hope that readers are now aware of these potential problems and that they explore whether the parameter values of interest can yield unrealistic predictions under a given software implementation.

**Fig. 9:**
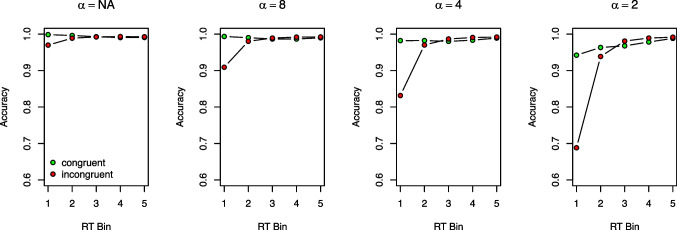
Illustration of CAFs for different values of $\alpha$. The *left-most panel* shows DMC’s predicted CAF without variability of the starting point being included; for the remaining three panels, starting points are taken from a symmetric Beta distribution with shape parameter $\alpha$ spanning the area between the boundaries. The influence of the starting point variability is particularly strong for incongruent trials. The other parameters were $\mu _c = 4$, $A = 0.1$, $b = 0.6$, $\sigma = 1$, and $\Delta t = 0.001$

### Fast errors and variability of the starting point

At the beginning of this article, we mentioned that CAFs in conflict tasks exhibit a typical pattern: While accuracy is high and rather independent of RT bin in congruent trials, it increases with increasing RT bins in incongruent trials. In other words, fast errors occur in incongruent trials. While the standard DM can only account for fast errors with the addition of starting point variability, DMC partially predicts such a pattern even without variability of the starting point (see the influence of *a*, $\tau$, and *A* on the CAFs in Fig. 5; and the left-most panel of Fig. 9). However, the empirical effect is often larger, and achieving a reasonable fit of the CAFs requires the inclusion of starting-point variability and thus of the parameter $\alpha$. As can be seen in Fig. 9, the smaller $\alpha$, the more pronounced fast errors become, because the variance of the underlying Beta distribution increases (see the Electronic Supplement B).

As an example, we fitted DMC to the Eriksen flanker and Simon data of Ulrich et al. (2015) and subsequently quantified the fit with the *RMSE* statistic. Without variability in the starting point, the misfit increased by about 4 ms on average. Additionally, compare Figs. 2 (DMC without starting point variability) and C1 (DMC with starting point variability) in Janczyk et al. (2025). There, it is apparent that the first RT bin of the incongruent CAF is systematically overestimated without variability in the starting point. Adding variability in the starting points thus improved the model fit.

In general, we recommend including starting point variability if it improves the model fit relative to the CAFs, although this parameter might not be of interest from a psychological perspective. Fitting DMC without this parameter in cases where the CAFs suggest its inclusion, likely introduces biases with respect to other model parameters (we come back to this in the next section, and see also the Supplementary Material of Evans & Servant, 2022, where DMC’s robustness to model misspecifications were explored).

### Summary and discussion

While the first applications of DMC relied on Monte Carlo simulations to approximate the PDF of RTs (e.g., Ulrich et al., 2015; see also the R package DMCfun by Mackenzie & Dudschig, 2021), methodological advances have since been made. In particular, efficient estimation via solving the Kolmogorov Forward Equation is promising, as is implemented in the R packages dRiftDM (Koob et al., 2025a) and |ream| (Hartmann & Murrow, 2024), or the Python package |pyBeam| (Murrow & Holmes, 2024b).

Most DMC parameters can be recovered (and thus are estimated) with reasonable reliability, except $\tau$ and *a*. The reasons are likely trade-offs between *a* and $\tau$. The corresponding time point of the maximum activation within the automatic route, $t_\text {max}$, can be recovered reliably, however. When fixing $a = 2$, recovery for *A* and $\tau$ seems to improve generally. In addition, estimating *a* freely can introduce numerical imprecision that may yield unrealistic model predictions. Fixing $a=2$ is one way to ensure that the derivative of the Gamma distribution function is always well-defined, thereby increasing numerical accuracy. In addition, $\alpha$ is recovered worse than ideal, but including starting point variability is sensible when fast errors are pronounced. In this case, DMC predicts fast errors easily, which likely prevents estimation biases in the remaining parameters.

Despite these insights and the methodological advances gained with respect to DMC, several open questions remain. For example, systematic investigations are missing concerning (a) the precise boundary conditions under which potential pitfalls of estimating *a* arise, (b) the potential harmful impact of particularly high accuracy for parameter recovery, or (c) the ranges under which $\tau$ is recovered well or not. Finally, especially with new software packages available, researchers have many degrees of freedom when estimating DMC, for example, with respect to (a) the approach and settings when deriving DMC’s predictions (i.e., Monte Carlo simulation vs. solving the Kolmogorov Forward Equation), (b) the optimization routine and the inference machine (e.g., classical vs. Bayesian estimation; Nelder–Mead with Grid Search vs. Differential Evolution), or (c) the choices about estimating or fixing parameters like $\alpha$ or *a*. Therefore, it will require more extensive simulation studies to provide clear guidelines on how best to estimate the parameters of DMC and how to design experiments that are particularly suited to this.

## Limitations, challenges, and outlook

Despite the widespread applications of DMC over the past 10 years, it certainly comes with limitations and challenges. Some of them are built into the model, and others have become apparent during its application. This section first covers such limitations, and we then proceed with general considerations of potential issues with DMC. For some of these points, future developments can be envisaged, which will be outlined as well. This section is therefore most interesting for readers with an interest in limits of DMC and modeling in general.

### Model architecture

Like virtually any stochastic model, DMC relies on auxiliary assumptions to derive testable predictions from its core postulates (see Ulrich, 2009). Consequently, it is conceivable that, while the core assumptions are empirically valid, the auxiliary assumptions may not be. In such cases, model predictions are typically not entirely accurate, although they may be approximately correct. For instance, DMC assumes that automatic activation follows a rescaled Gamma distribution function. By definition, this function cannot produce negative values, that is, it does not allow for undershooting of activation (see Koob et al., 2023b; Ulrich et al., 2015). Occurrence of negative values for the Gamma distribution function, as, for example, reflected in delta functions with negative values (i.e., reversed congruency effects), is the result of numerical inaccuracies (see Fig. 8). However, as also shown by Koob et al. , such auxiliary assumptions can be elaborated within the framework of DMC, and together with the core assumptions, the revised predictions can be analyzed.

A second point concerns the symmetric automatic activation caused by the irrelevant information in congruent and incongruent trials (see the left panel of Fig. 3). One might question whether this assumption is realistic. From a passive-decay perspective, it is reasonable to accept the idea that activation within the automatic route increases to a maximum and then decreases back to zero, regardless of whether the trial is congruent or incongruent. From an active suppression perspective, however, one may wonder whether suppression of automatic activation occurs equally in both types of trials. This concern has been raised by other authors as well. For example, Hübner and Töbel (2019, p. 9) note that “an optimal strategy would be to suppress irrelevant activation only for incongruent stimuli.”

Selective suppression has also been noted by Evans and Servant (2022) (see the Section “Neutral trials in conflict tasks” earlier in this article). To recap, these authors estimated the amplitude *A* separately for congruent and incongruent trials, and in most cases $A_\text {congruent}$ was close to zero and smaller than $A_\text {incongruent}$. This actually is the exact opposite to what Hübner and Töbel (2019) would suggest as an “optimal strategy.” At present, the best solution in this regard is unclear, and research on separating facilitation and interference with DMC is still rare (Evans & Servant, 2022; Smith & Ulrich, 2024, 2025). On the one hand, it is, of course, possible to estimate separate parameters and even implement different time courses for congruent and incongruent trials. On the other hand, an important question is then: How does the cognitive system know at the onset of response selection, that is, at $t=0$ of the diffusion process proper, whether the current trial is congruent or incongruent (for a specific example, see also Janczyk et al., 2025, in their critique of RDMC)? This issue might be less controversial when we assume the decision about the congruency of a trial and subsequent cognitive control mechanisms occurs in the course of response selection (e.g., during or after the amplitude of automatic processing reaches its maximum). This would require a continuous quantification of conflict during the course of a trial and an immediate adaptation of other parameters (as, e.g., implemented by López & Pomi, 2024; Weichart, Turner, & Sederberg, 2020).

A third point to mention here is that the standard version of DMC assumes a simultaneous onset of the relevant and the irrelevant stimulus feature. An earlier onset of distractors relative to the target is not uncommon though (Hübner & Töbel, 2019; Mattler, 2003; Weissman, Egner, Hawks, & Link, 2015). Indeed, the related conflict-like prime-probe paradigm involves the sequential presentation of a task-irrelevant prime stimulus followed by the task-relevant target or probe (e.g., Kunde & Wühr, 2006; Weissman, Schmidt, & Spinelli, 2024). Simulations provide a straightforward way to deal with such offsets in processing task-relevant and task-irrelevant features, and |DMCfun| already provides a parameter for doing so. The implementation in software packages like |dRiftDM| requires a bit more elaboration. While possible, one would have to calculate the expected time-course of processing within the automatic route and determine the corresponding activation upon the onset of target processing. Then, one can use this activation as the starting point of processing within the controlled route, while superimposing it with the activation within the automatic route.

Going even further, if irrelevant information is presented before the relevant information, it may serve as a warning signal, facilitating response speed (e.g., Bertelson & Tisseyre, 1968). Additionally, the delay between task-irrelevant and task-relevant processing provides time for the cognitive system to exploit the task-irrelevant information for efficient target processing (Logan & Zbrodoff, 1979). For example, according to the response modulation account in the proportion congruency effect literature (Weissman et al., 2024), participants identify the task-irrelevant feature before the task-relevant one, and use its identity to prepare a congruent response in mostly congruent blocks and/or an incongruent response in mostly incongruent blocks. Finally, the insertion of a delay between task-relevant and task-irrelevant stimulus features (e.g., flankers preceding the target) might give rise to attentional capture by the sudden onset of the target (e.g., Yantis & Jonides, 1984; Zhang, York, & Jonides, 2025). Hence, such a manipulation might not be as pure as it first seems, but rather introduce further processes and effects which are not incorporated into the standard version of DMC.

In summary, like all cognitive models, DMC is inherently limited in scope and cannot account for all phenomena within a research domain without introducing additional assumptions. Nevertheless, even in such cases, DMC may provide a heuristic tool for gaining insights into cognitive processing under conflict.

### Types of tasks and delta functions

As summarized at the beginning of the Section “Applications of DMC within the last 10 years,” DMC has been chiefly applied to the Eriksen flanker and Simon tasks. The few applications to Stroop tasks are indeed variations of Stroop tasks (such as the counting or the spatial Stroop task), or manual color-word Stroop tasks, except for an analysis presented in Appendix B of Kelber et al. (2025b). Why (vocal) Stroop tasks are rarely considered for modeling with DMC is something we can only speculate about. Of course, collecting manual responses is conceivably easier than collecting vocal responses, particularly in online experiments. Further, DMC may not fit the vocal Stroop data well, and there are reasons related to the Stroop task, as such, and to vocal responses.

First, Stroop tasks often come with four responses. Although accuracy-coded boundaries allow for inclusion of more than two stimuli and responses, DMs were designed for two choices/responses. Second, the time point where the automatic activation reaches its maximum, $t_{\text {max}}$, seems to be estimated very late in the manual Stroop task (see Hedge et al., 2022). If it is reached late relative to the time when the boundary *b* is exceeded, the automatic activation no longer follows a pulse-like time course. Rather, it is a monotonic, but more linear function (a similar argument has been made by Koob et al., 2023a, 2025b, in the context of dual-tasking). Matching with this rather monotonic influence of the automatic process, Pratte et al. (2010) reported delta functions from (manual, spatial) Stroop tasks that appear rather steep, compared with the typical delta functions obtained with Eriksen flanker or Simon tasks. In this sense, the time course of automatic activation assumed in DMC might not be valid for the Stroop task. Actually, a simple DM with different drift rates for congruent and incongruent conditions can predict a steep, linear delta function and might thus be preferred for Stroop tasks. Interestingly, such steep delta functions might be predicted by RDMC and DREAM, when assuming no change in the task-irrelevant processing. This is because, in this case, a model with a time-independent, constant drift rate results (i.e., a variant of the standard DM). Apart from these points, a late $t_{\text {max}}$ also likely introduces issues concerning parameter reliability, because, as we noted previously, the influences of $\tau$ and *a* on DMC’s predictions diminish when their values increase (see also Evans & Servant, 2022; Koob et al., 2025b). Third, although there is no principled reason for why vocal RTs are entirely special, response modality seems to make a difference in Stroop tasks (see, e.g., Parris, Hasshim, Wadsley, Augustinova, & Ferrand, 2022). Fennell and Ratcliff (2019) used a variant of a DM-like model to compare manual and vocal color-naming Stroop tasks (see in particular their Exp. 3) and concluded that “differences in conflict processing between the vocal response and manual response tasks in parts lies outside the decision-making process” (p. 2116). However, a direct comparison of manual and vocal Stroop tasks in terms of delta functions and DMC parameters is lacking.

Other tasks to which DMC may be applied include Navon’s local-global task (Navon, 1977) or priming tasks as are sometimes used in the context of conflict tasks. With the latter tasks, the problem of different onsets of task-relevant and task-irrelevant stimulus features applies, as has been briefly discussed in the previous section.

What should be kept in mind, however, is the shape of the delta function being predicted by DMC. Due to the pulse-like form of the expected automatic activation, DMC often predicts a non-linear delta function for reasonable and reliable parameter ranges (see Fig. 5). Even in the case where the delta function is positively sloped, its slope becomes less with longer RTs and often tilts downward at the longest RTs (i.e., the delta function slightly decreases again). Eriksen flanker tasks are indeed the typical ones, exactly producing such a positively sloped delta function, which explains, at least in parts, why this task (plus the Simon task) is one where DMC is predominantly applied to. An exception can be seen in Evans and Servant (2020), where the delta functions of an Eriksen flanker task data set appears rather steep and linear and qualitatively not fitting the data well. However, DMC has been fitted to the same data set in other studies (Janczyk et al., 2025; Ulrich et al., 2015), where the fit was much better, and where it predicted a more typical delta function.

### Number of parameters and their estimation

Another obvious limitation is that DMC involves model parameters whose values are unknown and must be estimated from empirical data. Parameter estimation typically becomes worse the more parameters must be estimated. In its full form, including starting point variability, DMC requires 8 parameters (see Table 1), whereas fixing the shape parameter to $a=2$ reduces it to 7 parameters. For specific research questions, further extensions of DMC have been proposed, which inherently increase the number of parameters (see, e.g., Evans & Servant, 2022; Koob et al., 2023b; Smith & Ulrich, 2024). Although other models (e.g., Heuer et al., 2023; Lee & Sewell, 2024) do involve even more parameters, it is clear that more parsimonious models are desirable. Parameter estimation can be challenging for optimization algorithms and often trade-offs appear between model parameters, the more complex the model is. As outlined in the section “Trial numbers and parameter recovery”, the parameter $\tau$ is particularly troublesome, especially with small trial numbers and when estimating *a* simultaneously (Janczyk et al., 2025; White et al., 2018). This might also be one reason why researchers interested in cognitive control and conflict tasks sometimes opt for simpler cognitive models, like the standard DM, even though these simpler models cannot adequately account for the underlying mechanisms in conflict tasks (see, e.g., Rey-Mermet et al., 2025, for a discussion). However, resorting to simpler models might hinder progress in gaining a better understanding of these unobservable mechanisms in conflict tasks.

**Fig. 10:**
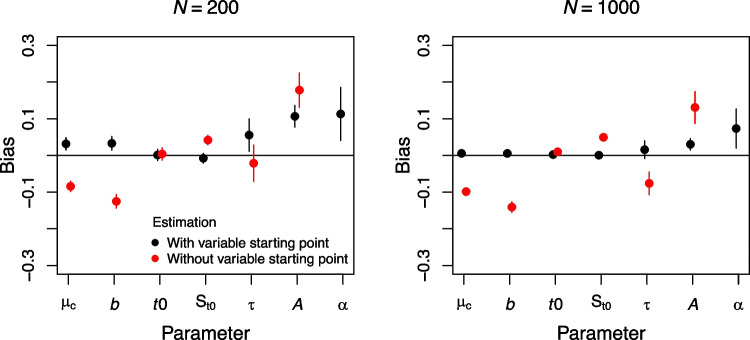
Effect of omitting $\alpha$ on biases of DMC parameters. The *left and right panels* show biases in parameter estimates when the simulated data sets comprised $N = 200$ and $N = 1000$ trials per congruency condition, respectively. For each simulated data set, we assumed variability in the starting point. *Black circles* show biases when parameters were estimated with variability in the starting point (i.e., correct model specification). *Red circles* show biases when parameters were estimated without variability in the starting point (i.e., model misspecification)

If parameters of a model cannot be estimated with reasonable precision, this usually reveals a lack of evidence about the postulated unobservable processes. To compensate for this, one has to (a) collect more and more data to provide enough information to guide parameter estimation and (b) use more elaborate parameter estimation approaches. One possible way to increase estimation precision is to constrain the parameter space. Bayesian approaches are promising in this regard. By using a combination of informative prior distributions and hierarchical estimation procedures, they help to constrain troublesome parameters like $\tau$ to reasonable values. For example, researchers modeling a Simon task, can try to keep $\tau$ within the typical range of 30 ms to 120 ms and avoid that $\tau$ takes on particularly unrealistic values for some participants. A systematic study that assesses the benefit of such a more advanced modeling approach is, however, not yet available.

Another approach that leans toward collecting more data could be to include additional experimental manipulations known to affect parameters of the model that are recovered well (such as *t*0). Although speculative, these additional conditions might help constrain parameters of DMC that are more difficult to recover (such as $\tau$) by forcing them to be equal across multiple conditions. Conceptually, such an approach would be similar to the use of filler items in clinical research, which can improve the estimation of simpler DMs (White et al., 2010b).

Finally, another potential way to increase the reliability of DMC’s parameters could be joint-modeling approaches, where not only choice RT data, but also neurophysiological data are used to guide parameter estimation (see Nunez, Fernandez, Srinivasan, & Vandekerckhove, 2024, for an introduction). In the context of DMC, Servant et al. (2016) have provided first steps in this direction by linking EEG and EMG components to DMC’s parameters. Still, more research is needed to explore these links. If it would be possible to use neurophysiological data to inform specific parameters of DMC directly, this would make the identification of the remaining parameters substantially easier.

In the section “Trial numbers and parameter recovery” we already mentioned that the addition of $\alpha$, the shape parameter of the Beta distribution defining the starting point distribution, might help in avoiding biased estimates in the case where CAFs show marked signs of fast errors (i.e., when the data-generating process may indeed involve variability in the starting point). However, most parameter recovery studies have not systematically examined the consequences of such a model misspecification, that is, the impact of not estimating $\alpha$ even though one should. The only exception is provided in the Supplementary Material of Evans and Servant (2022), but the authors did not quantify the biases (only reduced correlations between original and recovered parameters were reported).

To address this, we conducted a small recovery study with $N = 200$ and $N = 1000$ trials per congruency condition. Data were generated with variability in the starting point, and parameters were recovered both with and without such variability. The simulation setup and search space followed Janczyk et al. (2025). Figure 10 summarizes the main results. When the estimated model matched the model underlying the data (black points), biases were present but moderate. The largest biases were observed for *A* and $\alpha$ (with an overestimation of about $10\%$ for $N = 200$ and about $3\%$–$8\%$ for $N = 1000$). Importantly, dropping $\alpha$ introduced systematic biases (red points). While $\mu _c$, *b*, and $\tau$ were markedly underestimated, *A* and $S_{t0}$ were markedly overestimated. Therefore, we recommend including $\alpha$ whenever CAFs show a strong tendency for fast errors, even though recovery of $\alpha$ itself is relatively poor (with $r < .57$ in our recovery study).

### Summary

Despite some limitations and challenges, DMC has been successfully applied across various research fields. Certain issues relate to its architecture, such as symmetric activation in congruent and incongruent trials, and the simultaneous onset of task-relevant and task-irrelevant stimulus features. Others involve parameter estimation, with $\tau$ currently being the most challenging parameter. Addressing these challenges, for instance, through architectural extensions or improved estimation methods, remains a promising area for future research. Additionally, exploring DMC’s application to other tasks and systematically comparing its effectiveness–particularly in (vocal) Stroop tasks–represents an exciting opportunity for ongoing investigation.

## Conclusion

As outlined in this article, DMC has been applied across a wide range of cognitive psychology and, to a lesser extent, other fields of psychological research. One reason for this may be that the initially limited software solutions directly implementing DMC constrained its broader use. This has changed, however, with the simulation-based DMCfun R package (Mackenzie & Dudschig, 2021) and the recent R and Python packages dRiftDM (Koob et al., 2025a), ream (Hartmann & Murrow, 2024), and pyBeam (Murrow & Holmes, 2024b), which utilize the Kolmogorov Forward Equation to approximate the PDF of RTs without simulations (see also Richter et al., 2023). These developments substantially increase the accessibility of DMC across fields of psychological research.

It is also worth noting that DMC was explicitly developed to account for central phenomena observed in conflict tasks. This does not mean, however, that DMC can account for every empirical pattern, which is to be expected – after all, no model is ever complete. Relatedly, it has been claimed that DMC is inferior to other conflict models, particularly DSTP and SSP (Hübner et al., 2010; White et al., 2011), when fitted to Eriksen flanker task data (Evans & Servant, 2020). As mentioned earlier though, the delta functions predicted by DMC according to this study are not typical for DMC, and other studies found qualitatively better solutions for DMC (Janczyk et al., 2025; Ulrich et al., 2015). Yet, even if DMC were inferior, which might well be, partly because DMC is a relatively complex DM, it is important to note that DMC was not explicitly developed for Eriksen flanker tasks, but rather can be applied to other tasks as well, even those with negatively sloped delta functions, like the Simon task (see also Lee & Sewell, 2024; López & Pomi, 2024, for models capable of this). In this sense, we consider DMC a more general framework of processing architecture.

Despite its strengths, DMC also presents several interesting methodological challenges. Some parameters are inherently more demanding to recover, and several potential pitfalls – such as the extreme behavior of the Gamma distribution function’s derivative near $t = 0$ and possible trade-offs between parameters – are still being investigated. Consequently, although software implementations are available, a fully standardized practice for applying DMC has not yet emerged. The current lack of standardization, together with the scarcity of simulation studies, opens promising avenues for future methodological development. For example, clearer guidelines could help prevent suboptimal model applications and foster a more consistent interpretation of parameter estimates and model fit. Likewise, criteria for evaluating model fits would benefit from refinement. In some cases, fits have been labeled good despite systematic deviations (e.g., consistent over- or underestimation of the delta function), whereas other fits without apparent misfits have been described more cautiously. Establishing shared standards would thus support more reliable and transparent use of the DMC framework.

In sum, we believe DMC, as well as other mathematical models of human cognition, provides a powerful tool for fine-grained research into cognitive processes and their time-course. We hope this review will serve as a starting point for future research using DMC. It also hopefully provides guidance on DMC’s application, while highlighting open challenges that invite further work to deepen our understanding of its mathematical properties and potential pitfalls.

## Supplementary Information

Below is the link to the electronic supplementary material.

Supplementary file 1 (pdf 780 KB)

## Data Availability

No data have been collected.

## Footnotes

1 Although this description is rather standard, it certainly is an imprecise simplification. Indeed, there is no reason why the automatic route should not process target information to some degree, which might differ for the various conflict tasks though. A consequence of this reasoning might be, however, that more net activation in the automatic route exists in congruent than in incongruent trials.

2 While the mathematical details of a Brownian motion are not too important here, readers unfamiliar with it can think of it as a sum of (scaled) normal distributions that are accumulated for each time step $\Delta t$. Specifically, $\textbf{B}(t) = \sum _{i=1}^{t/\Delta t} \sqrt{\Delta t} \mathbf {Z_i}$, where all $\mathbf {Z_i}$ are independent standard normal distributions. Because the expected value of a Brownian motion is zero (i.e., the noise is unsystematic), the expected time course of the evidence accumulation process is just a straight line, that is, $\mathbb {E}[\textbf{X}(t)] = \mu t$.

3 Note that model predictions were derived in the unit of seconds and with a diffusion constant of $\sigma = 1$ for the present review. In the original publication by Ulrich et al. (2015), DMC was introduced in the unit of milliseconds and with $\sigma = 4$. This is just a matter of scaling and does not change the model behavior. We chose the scaling to align DMC more closely with the scaling of the standard DM. Nevertheless, readers only familiar with DMC (and not the standard DM), will find that parameter ranges are different. Equations on how to convert DMC’s parameters can be found in Koob et al. (2023b, Appendix B), and a function in the R programming language is provided at https://bit.ly/dmc_convert_prms.

4 dRiftDM runs DMC in the unit of seconds and with a diffusion constant of $\sigma = 1$, while DMCfun uses milliseconds and a diffusion constant of $\sigma = 4$, as in the original publication of Ulrich et al. (2015).

5 Parameter recovery refers to the ability to recover known parameter values when fitting a model to synthetic data generated from those very parameters. Ideally, the estimated parameters should closely match the values used to generate the synthetic data, which is a minimal prerequisite for judging parameters as reliable and interpretable. We will discuss this and typical estimation procedures in more detail in the later section “Technical aspects and recommendations”.

6 Note that these equations are a simplified version of the differential equations provided in the original article, and only hold in the unit of milliseconds with a step size of 1 ms.

7 The only parameter recovery was reported in the Supplementary Materials of the original publication. Here, however, only a few sets of parameters were recovered, and the number of trials used to generate the synthetic data was higher than one can expect from real data.

8 A further study by Schuch and Pütz (2021) has used a standard DM to investigate congruency sequence effects in task-switching and reported effects on drift rate and threshold. However, the standard DM cannot distinguish between controlled and automatic processing.

9 We use the notation of the original article here. The subscript ”L” in $\mu _{cL}$ stands for ”large”, because $\mu _{cL}>\mu _c$. The subscript ”S” in $\mu _{aS}(t)$ stands for ”small” with smaller values of *A* and $\tau$ compared with the values in $\mu _a(t)$. We thank Chunming Luo for clarifying this.

10 The conclusions of Kelber et al. (2025b) also match with results in Hübner and Töbel (2019, Exp. 3) who implemented a between-participants proportion congruency manipulation. Descriptively, a smaller amplitude of the automatic process was obtained as well.

11 More details on these aspects can also be found in our online book on DMs available at https://bit.ly/driftdm_workshop_book.

12 Note that the focus on the momentary change is also the reason why Eq. 10 requires the drift rates, that is, the first derivative of the expected time-course with respect to time *t* (see Eq. 3 and 5).

13 There are several ways to combine population members; the procedure described here is just one of many (Storn & Price, 1997). Additionally, Differential Evolution algorithms may not update all parameter values at once, but often select a random subset of parameters to update.

14 Unnormalized, because Bayes’ rule, the theorem underlying Bayesian inference, also includes the marginal probability of the data, *f*(*y*). If *y* is considered fixed, however, this marginal probability is constant and can be omitted for simplicity.

15 Note that the study by Servant et al. (2016) had an additional manipulation of the stimulus’ chroma (three levels).

16 The considered parameter search space and/or the starting values (in case the Nelder-Mead algorithm is used) must not be too narrow/close around the generating parameters. Otherwise, artificially high correlations can result and “successful” parameter recovery is an artifact of a highly constrained parameter search space (see Janczyk et al., 2025, for more information and a particular example).

17 This is what would happen, for example, if the Euler–Maruyama method (see Eq. 10) is used.

## Code availability

The code generating Fig. 10 is available at https://osf.io/yvrzs.
