# Supplementary material for: Ten years Diffusion Model for Conflict (DMC) tasks: Theoretical foundations, applications, practical recommendations, and open challenges
Source: Psychon Bull Rev. 2026 Apr 22;33(5):150. doi: 10.3758/s13423-026-02878-8 (PMC13102883; doi:10.3758/s13423-026-02878-8)
Supplement: Supplementary file 1 — (pdf 780 KB) [file 13423_2026_2878_MOESM1_ESM.pdf]

Electronic Supplement for the article:

Ten Years Diffusion Model for Conflict (DMC) Tasks: Theoretical Foundations, Applications, Practical Recommendations, and Open Challenges

Markus Janczyk[1], Ian G. Mackenzie[2], Rolf Ulrich[2], and Valentin Koob[1]

[1]University of Bremen, Bremen, Germany

[2]Eberhard Karls University Tübingen, Tübingen, Germany

Electronic Supplement A:
Interpretation of Standard DM Parameters

In the main text, we have covered interpretation of the standard DM parameters only briefly, but more information for unfamiliar readers is presented in the following.

The **boundary** b reflects how much evidence is needed to select a response, and it is interpreted as an indicator of how cautious (high b) or daring (low b) participants are. Changing b has opposite effects on RTs and error rates: Increasing b yields longer mean RTs but fewer errors. Hence, this parameter also captures differences in the speed-accuracy tradeoff settings (Lerche & Voss, 2018; Liesefeld & Janczyk, 2019, 2023). Speed versus accuracy instructions are a common means to induce a more liberal, daring versus a more conservative, cautious response style, which are then reflected by higher versus lower boundaries (e.g., Voss et al., 2004; see Mittelstädt et al., 2022, for a study with DMC). In the literature on aging, the boundary parameter helps explain (parts of) the prolonged RTs seen with older compared with younger adults (Ratcliff et al., 2000, 2001).

The **drift rate** μ is "the mean amount of information accumulated per unit of time" (Ratcliff & Rouder, 1998, p. 348) or, similarly, the "mean of the rate of information accumulation" (van Ravenzwaaij & Oberauer, 2009, p. 464). Consequently, a time-independent drift rate, such as μ_c in DMC, is often taken as measuring "the speed of information uptake and thus provides a measure of performance" (Voss et al., 2013, p. 387). A larger drift rate yields shorter RTs and fewer errors. That said, a multitude of task- and person-specific characteristics determines time-independent drift rates. First, it is "determined by the quality of evidence extracted from the stimulus or memory" (Ratcliff et al., 2016, p. 265) and it is larger for easy compared with more difficult tasks (Voss et al., 2004). However, intelligence has also been related to the drift rate, which typically takes a higher value with increasing intelligence (Lerche et al., 2020; Schmiedek et al., 2007; Schubert et al., 2016). In the context of DMC, the drift rate μ_c reflects the speed of controlled processing of the task-relevant stimulus feature, and one might say that μ_c

measures the efficiency of controlled stimulus-to-response translation.

The **non-decision time** $t0$ and its standard deviation S_{t0} represent the duration (and variability) of more peripheral processes, that is, "encoding the evidence from a stimulus that will drive the decision process, extracting the dimension(s) of the stimulus that form the basis of the decision from the stimulus or memory, and executing a response" (Ratcliff et al., 2016, p. 261). Increasing the non-decision time and its variability increases the mean and the variance of RTs, but does not affect error rates, as these parameters do not interact with the decision process. The variability parameter S_{t0} is often less important than $t0$ and its primary purpose is to improve the model fit (Boehm et al., 2018). Empirically, more complex responses result in longer non-decision times (e.g., Voss et al., 2004), although, for example, switching between tasks, also affects the $t0$ parameter (Schmitz & Voss, 2012). The diversity of manipulations affecting non-decision time has also given rise to objections regarding whether non-decision time, as understood in most models, indeed captures merely peripheral perceptual and motor processes outside the central decision process, and whether the two processes can be clearly distinguished. That said, there are suggestions in the literature to distinguish a motor boundary from a response boundary (Servant et al., 2021). The reasoning behind this is that motor activity in the form of EMG can be recorded prior to when the DM boundary is exceeded. This implies that some motor activity begins during what is classically attributed to the decision stage (see also Servant et al., 2015, for this idea in the context of conflict tasks). Furthermore, Bompas et al. (2025) provide a comprehensive review and theory of how to measure non-decision time empirically and relate this measure to model-estimated non-decision times. These authors also raise concerns that parts of the processes often attributed to the non-decision time might actually be related to a cascaded decision process, and that many cognitive models fail to capture this accurately. At present, though, and in the absence of more evaluation, DMC sticks with the standard interpretation of the non-decision time, and, by implication, to that of the decision time.

The **starting point** variability parameter α (see also Electronic Supplement B) reflects random decision biases that are present even before the actual decision process begins. From a psychological perspective, this is plausible if we assume that participants sometimes expect a particular stimulus and response prior to stimulus onset. However, since the specific stimulus is rarely known in advance, this bias remains unsystematic, leading to correct initial guesses on some trials (biases toward the upper boundary) and incorrect ones on others (biases toward the lower boundary). Despite its psychological plausibility, trial-by-trial variability in the starting point is usually not of central theoretical interest (Boehm et al., 2018; Lerche & Voss, 2016). Instead, it is primarily used to predict fast errors as often seen in incongruent conditions of conflict tasks. Thus, it often improves model fit and removing starting point variability typically impairs DMC's ability to fit data of conflict tasks (see, e.g., Janczyk et al., 2025).

Electronic Supplement B:

The Beta Distribution used for Starting Point Variability

When adding variability of the starting point in DMC, the starting points are typically drawn from a Beta distribution. This distribution is centered around 0, stretched to the boundary range $[-b, b]$, and has both shape parameters defined as α. Specifically, the distribution is defined as

$$f(x) = \frac{[(x - b_1)(b_2 - x)]^{\alpha-1}}{B(\alpha, \alpha)(b_2 - b_1)^{2\alpha-1}}$$

where $B(\alpha_1, \alpha_2)$ is the Eulerian Beta function

$$B(\alpha_1, \alpha_2) := \int_0^1 x^{\alpha_1 - 1}(1 - x)^{\alpha_2 - 1} dx \,,$$

and $\alpha_1 = \alpha_2 = \alpha$ in our special case (see also Ulrich et al., 2015, Appendix D).[1] Several examples are visualized in Figure S1. Increasing the parameter α, decreases the variance of the Beta distribution. Note that the exact distribution from which starting values are drawn seems not to affect model predictions much, at least for the standard DM (see also Ratcliff, 2013).

[1] In R, the values of the Beta distribution as a function of $x \in \{0, \ldots, 1\}$ are given by `dbeta(x, shape1, shape2)`, while the value of the Beta function is given by `beta(shape1, shape2)`.

Figure S1

Illustration of (General) Beta distributions.

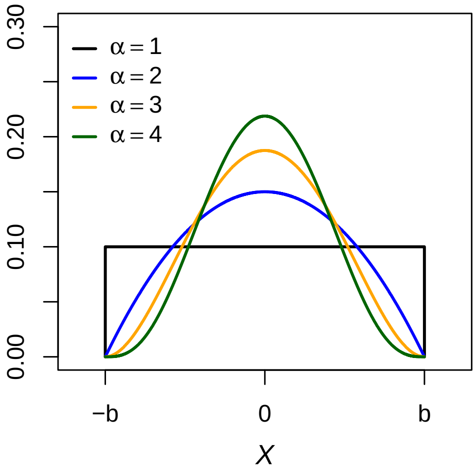

Note. The figure visualizes several general Beta distributions with both shape parameters set to α and stretched to an interval $[-b, b]$, from which a random starting point would then be drawn (see the right-most panel of Fig. 3 in the main text).

Electronic Supplement C:
Neutral Trials and a Time-Dependent Controlled Drift Rate

Smith and Ulrich (2024) proposed a DMC variant where the controlled process is time-dependent. Specifically, the time-dependent drift rate follows an exponential decay function

$$\mu_c(t) = \mu_c e^{-ct} ,$$

so that the corresponding expected time course of controlled processing is concave. Such a function implies that the rate of accumulation decreases over time and the longer the decision process runs, the less evidence is accumulated. It was speculated that with such a time-dependent controlled process, DMC can predict various R values (i.e., it can predict larger facilitation and larger interference effects). However, the authors did not report any model simulations.

We thus present a small simulation study, where we implemented this DMC variant with a time-dependent drift rate. For this, we used parameters typical for the Eriksen flanker and the Simon task: $\mu_c = 4$, $b = 0.5$, $t0 = 0.3$, $S_{t0} = 0.02$, $\tau \in \{0.04, 0.12\}$, $a = 2$, and $\alpha = 4$ (the time domain was in seconds and $\sigma = 1$). The parameter A was set to 0.1, 0, and -0.1 for the congruent, neutral, and incongruent condition. For the newly introduced parameter, we chose $c = 0$ (which results in standard DMC) and $c = 7$ (which results in a concave controlled process). Figure S2 shows the expected time-courses of controlled and superimposed processing (cf. Smith & Ulrich, 2024). The predicted R value is presented within each panel and listed in Table S1, along with more descriptive information.

As is evident, DMC predicted $R > 1$ for $\tau = 0.04$ and $R = 1.06$ for $\tau = 0.12$ if $c = 0$. Importantly, however, this latter prediction did not qualitatively change with $c = 7$, where $R = 1$.

Figure S2

Illustration of Controlled and Superimposed Processing for a DMC Variant With Concave Controlled Processing.

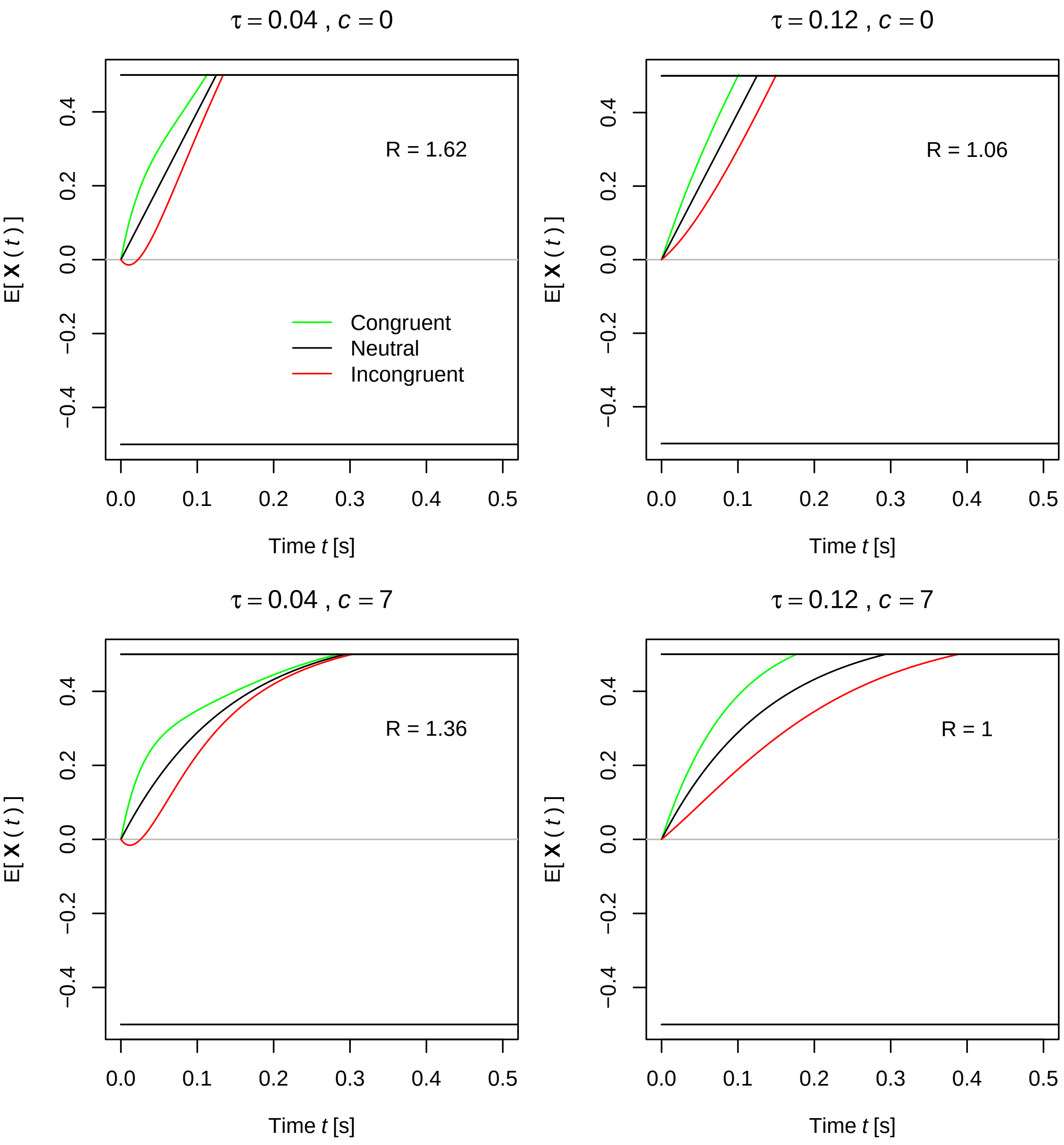

Note. The predicted R values (based on correct RTs) are shown within each panel. $R > 1$ equals a larger facilitation effect; $R < 1$ equals a larger interference effect; $R = 1$ equals comparable facilitation and interference.

Table S1

Summary of Model Predictions

τ	c	$M_{\text{congruent}}$	M_{neutral}	$M_{\text{incongruent}}$	R
0.04	0	0.403	0.416	0.424	1.62
0.04	7	0.430	0.449	0.463	1.36
0.12	0	0.399	0.416	0.432	1.06
0.12	7	0.424	0.449	0.474	1

Note. This table summarizes the predicted mean correct RTs (M) for the DMC variant with a concave controlled process as proposed by Smith and Ulrich (2024).

Electronic Supplement D:
Details on Summary Statistics

In the main text, we have only briefly mentioned summary statistics used in modeling, as they are not specific to DMC. For readers not familiar with them, we provide a detailed introduction in the context of DMC here.

The *Root Mean Squared Error* ($RMSE$) statistic is similar to the common least-squares method (as, e.g., used in linear regression), and it compares the squared difference between observed and predicted quantiles of correct RTs, and the squared difference between observed and predicted CAFs for each condition, that is,

$$MSE_{\text{CAF}} = \frac{1}{KC} \sum_{k=1}^{K} \sum_{c=1}^{C} \left[CAF_{\text{pred},k,c} - CAF_{\text{obs},k,c}\right]^2$$

and

$$MSE_{\text{RT}} = \frac{1}{KJ} \sum_{k=1}^{K} \sum_{j=1}^{J} \left[Q_{\text{pred},k,j} - Q_{\text{obs},k,j}\right]^2 .$$

Here, CAF and Q stand for the CAF and quantile values (i.e., accuracy and [correct] RTs, respectively; see also Fig. 2 in the main text). K indicates the number of congruency conditions (typically $K = 2$ for DMC), C indicates the number of bins used for the CAF (often $C = 5$), and J indicates the number of quantile levels (often $J = 9$ or $J = 19$). Finally, the subscripts *pred* and *obs* indicate predicted and observed data. The final $RMSE$ statistic is then a weighted combination after taking the root,

$$RMSE = w_{\text{CAF}} \cdot \sqrt{MSE_{\text{CAF}}} + w_{\text{RT}} \cdot \sqrt{MSE_{\text{RT}}} .$$

Ulrich et al. (2015) proposed setting the weights to

$$w_{\text{RT}} = \frac{KJ}{K(J+C)} \quad \text{and} \quad w_{\text{CAF}} = 1500(1 - w_{\text{RT}}) ,$$

meaning that a prediction error of 1% for the CAFs corresponds to a prediction error of

$0.01 \cdot 1500 = 15$ ms (note that this requires (a) quantile values to be in the unit of milliseconds, and (b) CAFs to express relative frequencies ranging between 0 and 1).

The χ^2 and G^2 statistics are based on a comparison of expected and observed proportions across bins. The former is the classical χ^2 statistic proposed by Pearson (1900), while the latter is an alternative formula based on likelihood theory. In fact, G^2 can be described as a likelihood ratio χ^2 statistic (Kendall & Stuart, 1961). Although both are derived from different perspectives and have slightly different properties (Cressie & Read, 1989), they are equivalent for large sample sizes and behave very similarly when fitting a model.

To derive the respective statistic, one first calculates quantiles for the observed data (e.g., the .1, .3, .5, .7, and .9 quantiles; Ratcliff & Tuerlinckx, 2002) for both correct and incorrect RTs across conditions, thereby creating cutoff values to bin data. During the estimation procedure, the simulated data is then binned using these observed quantile values, leading to predicted proportions of RTs in each bin. These predicted proportions are then compared with the observed proportions (with the latter inherently defined by the observed quantiles). The χ^2 statistic is calculated as (e.g., White et al., 2018)

$$\chi^2 = \sum_{k=1}^{K} N_k \sum_{b=1}^{B} \frac{(p_{kb} - \pi_{kb})^2}{\pi_{kb}} \,,$$

where K refers to the number of congruency conditions (typically $K = 2$ for DMC) and B to the number of bins for both correct and incorrect responses (e.g., B is 12 when using 6 bins for correct and incorrect responses). N_k is an individual's number of trials per congruency condition and p_{kb} and π_{kb} are the observed and predicted proportions of trials in bin b of condition k, respectively. G^2 uses essentially the same information as χ^2 does, but is slightly differently calculated as

$$G^2 = 2 \sum_{k=1}^{K} \sum_{b=1}^{B} N_k p_{kb} \log\left(\frac{p_{kb}}{\pi_{kb}}\right) .$$

Note that both p_{kb} and π_{kb} must sum to 1 across the B bins, that is,

$$\sum_{b=1}^{B} p_{kb} = \sum_{b=1}^{B} \pi_{kb} = 1 \qquad \forall k \in \{1, ..., K\} .$$

Given that there are fewer incorrect than correct responses by nature, particularly for the congruent condition, the number of bins for incorrect responses might be smaller than the number of bins for correct responses. For example, White et al. (2018) used only the median RT for incorrect responses when the respective number was less than or equal to 5, three RT quantiles (.3, .5, .9) when the number of errors was larger than 5, but less than or equal to 10, and otherwise five RT quantiles (.1, .3, .5, .7, .9). An alternative to adapting the number of bins for error data is to use CAFs by plugging in the observed and predicted proportions of error trials for p_{kb} and π_{kb}, so that B results from the number of bins based on correct quantiles and the number of bins of the CAFs. This approach was originally proposed by Hübner (2014) and subsequently adopted by others, especially in combination with the G^2 statistic and DMC (C. Luo & Proctor, 2022; J. Luo et al., 2023; Mahani et al., 2019; Servant et al., 2016; White et al., 2018).

An potential advantage of the original χ^2 and G^2 over the $RMSE$ is that they follow approximately χ^2 distributions with $df = K(B-1) - M$, where M is the number of parameters being estimated (for small cell frequencies, the approximation to the χ^2 distribution is better for G^2 statistic; Cochran, 1952). Thus, after having fit DMC, one could test whether the model predictions deviate significantly from the observed data. However, we are not aware that this has been done in practice, probably because, like any statistical test, the test of equivalence between observed and predicted data tends to be overly sensitive for larger sample sizes. Additionally, researchers are usually satisfied with a good qualitative fit between observed and predicted data.

Another commonly used summary statistic, Λ, is based on the negative log-likelihood of observed RT quantile proportions, assuming a multinomial density function (Hedge et al.,

2019, 2020, 2022; see also Appendix B of Vandekerckhove & Tuerlinckx, 2007, and Brown & Heathcote, 2003). It is closely related to the G^2 statistic, and both differ only by a constant for any given dataset and is calculated as

$$\Lambda = -2 \sum_{k=1}^{K} \sum_{b=1}^{B} N_k p_{kb} \log\left(\pi_{kb}\right) \, .$$

Note that, because G^2 and Λ originate from likelihood theory, they can be used to derive the Akaike and Bayesian Information Criteria (Akaike, 1973; Schwarz, 1978). Specifically, AIC is computed as

$$AIC = G^2 + 2M = \Lambda + 2M \, ,$$

while BIC is calculated as

$$BIC = G^2 + M \log(N) = \Lambda + M \log(N) \, ,$$

with N being the number of all observations for an individual.

For completeness, a final summary statistic in the context of DMC was used by Hübner and Pelzer (2020) and named the *Squared Percentage Error* (SPE). It is conceptually similar to the $RMSE$, as it directly compares summary statistics obtained from the observed and predicted data:

$$SPE = \sum_{k=1}^{K} \sum_{j=1}^{J} \left(\frac{\mathrm{Obs}_{kj} - \mathrm{Pred}_{kj}}{\mathrm{Pred}_{kj}} \right)^2 \, .$$

Here, Obs_{kj} and Pred_{kj} refer to any summary statistics obtained from the observed or predicted data, respectively. For example, Hübner and Pelzer (2020) used five quantile values of correct RTs, five accuracy values obtained from a CAF, and five mean RT values within each CAF interval (per congruency condition).

Electronic Supplement E:
Details on Maximum Likelihood Estimation

Instead of using DMC's predicted RT data by converting them into quantiles and CAFs, one can also directly use the (full) predicted distribution of RTs. Specifically, for every parameter set θ, DMC predicts probability density functions (PDFs), $f_{r,k}(y|\theta)$, indicating the probability to observe certain (ranges of) RTs for a specific response type r (e.g., correct or incorrect) and congruency condition k. Note that there is one PDF for each condition, and that each PDF integrates to one across both response types r. In the context of DMC, θ is a vector containing DMC's parameters (e.g., μ_c, A, τ, etc.). Because the predicted PDFs provide us with information about how probable it is to observe certain RTs given some set of parameters, we can use them to obtain those parameters under which the observed data becomes most likely. This approach is called *Maximum Likelihood Estimation (MLE)*, and it is one of the most widely used and principled concepts in statistics; for example, since MLE reaches asymptotically the lower bound of the Cramér-Rao-inequality.

In detail, given a set of observed and independent RTs for a single individual, which we denote as $y_{r,k}$, we can write the likelihood of the parameters given the observed data as

$$L(\theta \mid y) = f(y \mid \theta) = \prod_k \prod_r f_{r,k}(y_{r,k}|\theta)\,.$$

The right part is the product of PDF values for each observed RT in a data set, given a set of parameters θ. If we treat the data as fixed and the parameters as variable, this expression is referred to as the likelihood function $L(\theta \mid y)$. It essentially indicates how likely it is to observe the data for different sets of parameters. The higher its value, the more likely it is that the data were generated with these parameters, and when fitting DMC, we aim to find the parameter set that maximizes the likelihood. In practice, however, most model-fitting algorithms are designed to minimize by default. Additionally,

since the likelihood function is a product of many terms, each typically smaller or larger than 1, it can yield extremely small or large values, leading to numerical problems. To address both issues, one typically minimizes the negative log-likelihood, $-\log(L(\theta \mid y))$. Note that $L(\theta|y)$ has nothing to do with formal probabilities. It also does *not* indicate the probability of the parameters given the data, which is called the posterior probability and is the core quantity used for Bayesian estimation.

While MLE is a common method for fitting the standard DM (Voss et al., 2015), it has rarely been used for DMC. As far as we are aware, only Janczyk et al. (2025) and Koob et al. (2023) have applied it so far. One reason is that, until recently, DMC predictions were predominantly obtained with Monte Carlo simulations. Consequently, the predicted data obtained from the simulation were RT data and not directly the PDFs. Of course, one can approximate the PDFs from the simulated RTs via kernel density estimation (Evans & Servant, 2020, 2022; Turner & Sederberg, 2014).

Electronic Supplement F:

Details on the Derivative of the Gamma Distribution Function

To derive Equation 13 as presented in the main text, we start with the first derivative of the (rescaled) Gamma distribution function as used by Ulrich et al. (2015):

$$\mu_a(t) = Ae^{-\frac{t}{\tau}} \cdot \left[\frac{te}{(a-1)\tau}\right]^{a-1} \cdot \left[\frac{a-1}{t} - \frac{1}{\tau}\right] .$$

We extend and then split the first parenthesis using basic rules for exponents:

$$\mu_a(t) = Ae^{-\frac{t}{\tau}} \cdot \left[\frac{te}{(a-1)\tau}\right]^{a-2+1} \cdot \left[\frac{a-1}{t} - \frac{1}{\tau}\right]$$

$$= Ae^{-\frac{t}{\tau}} \cdot \left[\frac{te}{(a-1)\tau}\right]^{a-2} \cdot \left[\frac{te}{(a-1)\tau}\right] \cdot \left[\frac{a-1}{t} - \frac{1}{\tau}\right] .$$

Finally, we join the last two parenthesis and simplify the expression:

$$\mu_a(t) = Ae^{-\frac{t}{\tau}} \cdot \left[\frac{te}{(a-1)\tau}\right]^{a-2} \cdot \left[\frac{te}{(a-1)\tau}\right] \cdot \left[\frac{a-1}{t} - \frac{1}{\tau}\right]$$

$$= Ae^{-\frac{t}{\tau}} \cdot \left[\frac{te}{(a-1)\tau}\right]^{a-2} \cdot \left[\frac{te(a-1)}{(a-1)\tau t} - \frac{te}{(a-1)\tau^2}\right]$$

$$= Ae^{-\frac{t}{\tau}} \cdot \left[\frac{te}{(a-1)\tau}\right]^{a-2} \cdot \left[\frac{e(a-1)}{(a-1)\tau} - \frac{te}{(a-1)\tau^2}\right]$$

$$= Ae^{-\frac{t}{\tau}} \cdot \left[\frac{te}{(a-1)\tau}\right]^{a-2} \cdot \left[\frac{\tau e(a-1)}{(a-1)\tau^2} - \frac{te}{(a-1)\tau^2}\right]$$

$$= Ae^{-\frac{t}{\tau}} \cdot \left[\frac{te}{(a-1)\tau}\right]^{a-2} \cdot \left[\frac{\tau e(a-1) - te}{(a-1)\tau^2}\right] .$$

References

Akaike, H. (1973). Maximum likelihood identification of Gaussian autoregressive moving average models. *Biometrika*, *60*(2), 255–265. https://doi.org/10.1093/biomet/60.2.255

Boehm, U., Annis, J., Frank, M. J., Hawkins, G. E., Heathcote, A., Kellen, D., Krypotos, A.-M., Lerche, V., Logan, G. D., Palmeri, T. J., et al. (2018). Estimating across-trial variability parameters of the diffusion decision model: Expert advice and recommendations. *Journal of Mathematical Psychology*, *87*, 46–75. https://doi.org/10.1016/j.jmp.2018.09.004

Bompas, A., Sumner, P., & Hedge, C. (2025). Non-decision time: The Higgs Boson of decision. *Psychological Review*, *132*(2), 330–363. https://doi.org/https://doi.org/10.1037/rev0000487

Brown, S., & Heathcote, A. (2003). QMLE: Fast, robust, and efficient estimation of distribution functions based on quantiles. *Behavior Research Methods, Instruments, & Computers*, *35*, 485–492. https://doi.org/10.3758/BF03195527

Cochran, W. G. (1952). The $\chi 2$ test of goodness of fit. *The Annals of Mathematical Statistics*, 315–345.

Cressie, N., & Read, T. R. (1989). Pearson's X^2 and the loglikelihood ratio statistic G^2: A comparative review. *International Statistical Review*, *57*(1), 19–43. https://doi.org/10.2307/1403582

Evans, N. J., & Servant, M. (2020). A comparison of conflict diffusion models in the flanker task through pseudolikelihood Bayes factors. *Psychological Review*, *127*(1), 114–135. https://doi.org/10.1037/rev0000165

Evans, N. J., & Servant, M. (2022). A model-based approach to disentangling facilitation and interference effects in conflict tasks. *Psychological Review*, *129*(5), 1183–1209. https://doi.org/10.1037/rev0000357

Hedge, C., Powell, G., Bompas, A., & Sumner, P. (2020). Self-reported impulsivity does not predict response caution. *Personality and Individual Differences*, *167*, 110257. https://doi.org/10.1016/j.paid.2020.110257

Hedge, C., Powell, G., Bompas, A., & Sumner, P. (2022). Strategy and processing speed eclipse individual differences in control ability in conflict tasks. *Journal of Experimental Psychology: Learning, Memory, and Cognition*, *48*(10), 1448–1469. https://doi.org/10.1037/xlm0001028

Hedge, C., Vivian-Griffiths, S., Powell, G., Bompas, A., & Sumner, P. (2019). Slow and steady? Strategic adjustments in response caution are moderately reliable and correlate across tasks. *Consciousness and Cognition*, *75*, 102797. https://doi.org/10.1016/j.concog.2019.102797

Hübner, R. (2014). Does attentional selectivity in global/local processing improve discretely or gradually? *Frontiers in Psychology*, *5*, 61. https://doi.org/10.3389/fpsyg.2014.00061

Hübner, R., & Pelzer, T. (2020). Improving parameter recovery for conflict drift-diffusion models. *Behavior Research Methods*, *52*, 1848–1866. https://doi.org/10.3758/s13428-020-01366-8

Janczyk, M., Mackenzie, I. G., & Koob, V. (2025). A comment on the Revised Diffusion Model for Conflict tasks (RDMC). *Psychonomic Bulletin & Review*, *32*, 690–704. https://doi.org/10.3758/s13423-024-02574-5

Kendall, M. G., & Stuart, A. (1961). *The advanced theory of statistics, volume 2: Inference and relationship*. Hafner Publishing Company.

Koob, V., Mackenzie, I. G., Ulrich, R., Leuthold, H., & Janczyk, M. (2023). The role of task-relevant and task-irrelevant information in congruency sequence effects: Applying the diffusion model for conflict tasks. *Cognitive Psychology*, *140*, 101528. https://doi.org/10.1016/j.cogpsych.2022.101528

Lerche, V., von Krause, M., Voss, A., Frischkorn, G. T., Schubert, A.-L., & Hagemann, D. (2020). Diffusion modeling and intelligence: Drift rates show both domain-general and domain-specific relations with intelligence. *Journal of Experimental Psychology: General*, *149*(12), 2207–2249. https://doi.org/10.1037/xge0000774

Lerche, V., & Voss, A. (2016). Model complexity in diffusion modeling: Benefits of making the model more parsimonious. *Frontiers in Psychology*, 1324. https://doi.org/10.3389/fpsyg.2016.01324

Lerche, V., & Voss, A. (2018). Speed–accuracy manipulations and diffusion modeling: Lack of discriminant validity of the manipulation or of the parameter estimates? *Behavior Research Methods*, *50*, 2568–2585. https://doi.org/10.3758/s13428-018-1034-7

Liesefeld, H. R., & Janczyk, M. (2019). Combining speed and accuracy to control for speed-accuracy trade-offs (?) *Behavior Research Methods*, *51*, 40–60. https://doi.org/10.3758/s13428-018-1076-x

Liesefeld, H. R., & Janczyk, M. (2023). Same same but different: Subtle but consequential differences between two measures to linearly integrate speed and accuracy (LISAS vs. BIS). *Behavior Research Methods*, *55*(3), 1175–1192. https://doi.org/10.3758/s13428-022-01843-2

Luo, C., & Proctor, R. W. (2022). A diffusion model for the congruency sequence effect. *Psychonomic Bulletin & Review*, *29*(6), 2034–2051. https://doi.org/10.3758/s13423-022-02119-8

Luo, J., Yang, M., & Wang, L. (2023). Learned irrelevant stimulus-response associations and proportion congruency effect: A diffusion model account. *Journal of Experimental Psychology: Learning, Memory, and Cognition*, *49*(8), 1218–1246. https://doi.org/10.1037/xlm0001158

Mahani, M.-A. N., Bausenhart, K. M., Ahmadabadi, M. N., & Ulrich, R. (2019). Multimodal Simon effect: A multimodal extension of the diffusion model for conflict

tasks. *Frontiers in Human Neuroscience*, *12*, 507. https://doi.org/10.3389/fnhum.2018.00507

Mittelstädt, V., Miller, J., Leuthold, H., Mackenzie, I. G., & Ulrich, R. (2022). The time-course of distractor-based activation modulates effects of speed-accuracy tradeoffs in conflict tasks. *Psychonomic Bulletin & Review*, *29*(3), 837–854. https://doi.org/10.3758/s13423-021-02003-x

Pearson, K. (1900). On the criterion that a given system of deviations from the probable in the case of a correlated system of variables is such that it can be reasonably supposed to have arisen from random sampling. *The London, Edinburgh, and Dublin Philosophical Magazine and Journal of Science*, *50*(302), 157–175. https://doi.org/10.1080/14786440009463897

Ratcliff, R. (2013). Parameter variability and distributional assumptions in the diffusion model. *Psychological Review*, *120*(1), 281–292. https://doi.org/10.1037/a0030775

Ratcliff, R., & Rouder, J. N. (1998). Modeling response times for two-choice decisions. *Psychological Science*, *9*(5), 347–356. https://doi.org/10.1111/1467-9280.00067

Ratcliff, R., Smith, P. L., Brown, S. D., & McKoon, G. (2016). Diffusion decision model: Current issues and history. *Trends in Cognitive Sciences*, *20*(4), 260–281. https://doi.org/10.1016/j.tics.2016.01.007

Ratcliff, R., Spieler, D., & Mckoon, G. (2000). Explicitly modeling the effects of aging on response time. *Psychonomic Bulletin & Review*, *7*(1), 1–25. https://doi.org/10.3758/BF03210723

Ratcliff, R., Thapar, A., & McKoon, G. (2001). The effects of aging on reaction time in a signal detection task. *Psychology and Aging*, *16*(2), 323–341. https://doi.org/10.1037/0882-7974.16.2.323

Ratcliff, R., & Tuerlinckx, F. (2002). Estimating parameters of the diffusion model: Approaches to dealing with contaminant reaction times and parameter variability.

Psychonomic Bulletin & Review, *9*(3), 438–481. https://doi.org/10.3758/BF03196302

Schmiedek, F., Oberauer, K., Wilhelm, O., Süß, H.-M., & Wittmann, W. W. (2007). Individual differences in components of reaction time distributions and their relations to working memory and intelligence. *Journal of Experimental Psychology: General*, *136*(3), 414–429. https://doi.org/10.1037/0096-3445.136.3.414

Schmitz, F., & Voss, A. (2012). Decomposing task-switching costs with the diffusion model. *Journal of Experimental Psychology: Human Perception and Performance*, *38*(1), 222–250. https://doi.org/10.1037/a0026003

Schubert, A.-L., Frischkorn, G. T., Hagemann, D., & Voss, A. (2016). Trait characteristics of diffusion model parameters. *Journal of Intelligence*, *4*(3), 7. https://doi.org/10.3390/jintelligence4030007

Schwarz, G. (1978). Estimating the dimension of a model. *The Annals of Statistics*, *6*(2), 461–464. https://doi.org/10.1214/aos/1176344136

Servant, M., Logan, G. D., Gajdos, T., & Evans, N. J. (2021). An integrated theory of deciding and acting. *Journal of Experimental Psychology: General*, *150*(12), 2435–2454. https://doi.org/10.1037/xge0001063

Servant, M., White, C., Montagnini, A., & Burle, B. (2015). Using covert response activation to test latent assumptions of formal decision-making models in humans. *Journal of Neuroscience*, *35*(28), 10371–10385. https://doi.org/10.1523/JNEUROSCI.0078-15.2015

Servant, M., White, C., Montagnini, A., & Burle, B. (2016). Linking theoretical decision-making mechanisms in the simon task with electrophysiological data: A model-based neuroscience study in humans. *Journal of Cognitive Neuroscience*, *28*(10), 1501–1521. https://doi.org/10.1162/jocn_a_00989

Smith, P., & Ulrich, R. (2024). The neutral condition in conflict tasks: On the violation of the midpoint assumption in reaction time trends. *Quarterly Journal of Experimental Psychology*, *77*(5), 1023–1043. https://doi.org/10.1177/17470218231201476

Turner, B. M., & Sederberg, P. B. (2014). A generalized, likelihood-free method for posterior estimation. *Psychonomic Bulletin & Review*, *21*, 227–250. https://doi.org/10.3758/s13423-013-0530-0

Ulrich, R., Schröter, H., Leuthold, H., & Birngruber, T. (2015). Automatic and controlled stimulus processing in conflict tasks: Superimposed diffusion processes and delta functions. *Cognitive Psychology*, *78*, 148–174. https://doi.org/10.1016/j.cogpsych.2015.02.005

Vandekerckhove, J., & Tuerlinckx, F. (2007). Fitting the Ratcliff diffusion model to experimental data. *Psychonomic Bulletin & Review*, *14*, 1011–1026. https://doi.org/10.3758/BF03193087

van Ravenzwaaij, D., & Oberauer, K. (2009). How to use the diffusion model: Parameter recovery of three methods: EZ, fast-dm, and DMAT. *Journal of Mathematical Psychology*, *53*(6), 463–473. https://doi.org/10.1016/j.jmp.2009.09.004

Voss, A., Nagler, M., & Lerche, V. (2013). Diffusion models in experimental psychology. *Experimental Psychology*, *60*(6), 385–402. https://doi.org/10.1027/1618-3169/a000218

Voss, A., Rothermund, K., & Voss, J. (2004). Interpreting the parameters of the diffusion model: An empirical validation. *Memory & Cognition*, *32*, 1206–1220. https://doi.org/10.3758/BF03196893

Voss, A., Voss, J., & Lerche, V. (2015). Assessing cognitive processes with diffusion model analyses: A tutorial based on fast-dm-30. *Frontiers in Psychology*, *6*, 336. https://doi.org/10.3389/fpsyg.2015.00336

White, C. N., Servant, M., & Logan, G. D. (2018). Testing the validity of conflict drift-diffusion models for use in estimating cognitive processes: A

parameter-recovery study. *Psychonomic Bulletin & Review*, *25*(1), 286–301. https://doi.org/10.3758/s13423-017-1271-2
